# Performance of the DK and DKM shell elements in the standard, and the sequential, finite-element based limit analysis

**DOI:** 10.1038/s41598-025-33522-5

**Published:** 2026-04-13

**Authors:** Vítězslav Štembera

**Affiliations:** Institute for Mechanics of Materials and Structures, TU Wien, Karlsplatz 13, 1040 Vienna, Austria

**Keywords:** Finite element-based limit analysis, Sequential finite element-based limit analysis, Upper-bound formulation, Shell elements, DKMQ24, DKMQ24+, Engineering, Materials science, Mathematics and computing, Physics

## Abstract

This paper investigates the applicability of the DK (Discrete Kirchhoff) and DKM (Discrete Kirchhoff–Mindlin) shell element classes for the first time within the framework of the standard (and sequential) finite-element-based limit analysis, which is a direct method used for determining the plastic collapse (and post-collapse) behavior of structures. Despite these elements not being able to guarantee a strict upper bound, it is shown that they exhibit a significantly lower discretization error compared to the strict upper-bound formulated shell elements used in literature. The higher numerical efficiency of quadrangle elements over triangle elements is observed. The behavior of the tested shell elements on distorted meshes is also explored and discussed. The convergence orders of the tested shell elements are also estimated.

## Introduction

The prediction of the collapse load (limit load) of a structure made of a material exhibiting plastic behavior is often of practical interest. The standard approach to obtain such collapse loads is based on iterative elastoplastic analysis using classical nonlinear finite element methods. However, alternative approaches can be applied: the finite-element-based limit analysis (FELA) or the elastic-reduction method (see, e.g.^1^). The former approach is based on the limit theorems of plasticity, first formulated by A.A.Gvozdev in 1936 (The article *The determination of the value of the collapse load for statically indeterminate systems undergoing plastic deformation* by A.A. Gvozdev, written in Russian, appeared in a slim volume entitled *Proceedings of the Conference on Plastic Deformations, December* 1936, which was issued by the Academy of Sciences of the U.S.S.R. in 1938 under editorship of B.G.Galerkin^2^. English translation of this paper firstly appeared in 1960^3^) and later, independently, in 1952 by D.C.Prager^4^. A monotonically increasing external load is considered, precluding any local unloading. Thereby, the collapse load is obtained as the minimum of a certain convex optimization problem, either considering kinematically compatible velocity fields (upper bound approach) or statically admissible stress fields (lower bound approach) within the structure, at the time instant of collapse. FELA is a stable and numerically efficient approach compared to the standard scheme based on iterative, incremental elastoplastic analysis. High computational efficiency is further achieved by the fact, that the problem is formulated as the second-order cone programming (SOCP), for which highly efficient primal-dual interior-point optimizers have been developed in last decades^5^. Further advantages of FELA include the straightforward incorporation of multi-surface plasticity models—achieved by embedding all yield surfaces directly into the optimization problem, as interior-point methods can handle non-smooth yield surfaces^6^—as well as the straightforward inclusion of transverse shear stresses in the failure criterion for beam and shell structures (see, for example,^7^). However, FELA also has some principal disadvantages: it neglects the influence of material elasticity, assumes small deformations, and presumes ideally-plastic material behavior.

Moreover, it is important to keep in mind that FELA does not account for a loss of stability due to elastic buckling, which must be checked separately. The assumptions of small deformations and, to some extent, ideal-plasticity can be overcome by the so-called sequential finite-element-based limit analysis (SFELA), as, e.g., shown in^8–10^. In this process, FELA method is called repeatedly, with the geometry and plastic strain being updated after each iteration. Updating the plastic strain allows for the incorporation of non-decreasing hardening curves (with zero slope at infinity). However, incorporating softening results in a non-convex optimization problem of the MPEC type, which is numerically challenging to handle^11^. For details on this approach, often referred to as the extended FELA, see^12^.

In this paper we focus on the upper bound FELA approach applied on three-dimensional shell structures. Much attention in literature is dedicated to solid elements, plane-strain elements^13–17^, plate elements^7,18–26^ and axisymmetrical shells^10^. Surprisingly, shell elements, which are the most used finite elements in real engineering applications (From 110 real customer problems from an area of civil engineering published on websites of ADINA Structures^27^, ATENA^28^ and RFEM^29^ finite element programs, 66 projects (i.e., 60%) contain shell elements), seem not to obtain in the context of FELA a significant attention. Exception to the rule are papers by Bleyer^20^, Corradi^30^, Milani^31^ and Tr$\grave{\hat{\text {a}}}$n^32^. Bleyer^20^ uses a 6-node triangle shell element with straight sides, a quadratic approximation of velocities and a linear approximation of rotational velocities, equipped with discontinuities on element boundaries. Corradi^30^ uses continuous flat triangular shell elements called TRIC. Milani^31^ uses curved 6-node rigid triangular elements with discontinuities on element sides. Tr$\grave{\hat{\text {a}}}$n^32^ uses continuous flat quad Kirchhoff shell elements. Makrodimopoulos^25^ presents a class of upper bound triangular plane strain and Kirchhoff plate elements with straight sides based on the use of Bernstein polynomials. If certain restrictive conditions are met, then finite elements can be developed that guarantee a strict upper bound^20,30^, see also Section “Strict upper-bound versus approximative upper bound”. This numerically advantageous property, however, prevents the use of most standard shell elements in FEA—particularly quadrilateral elements or those incorporating cubic bubble functions—as they require numerical quadrature over the element, which does not yield any bounding. An example of the numerical effectiveness of triangular, quadrilateral, and hexagonal elements with bubble functions in in-plane strain limit analysis is given in^17^. Thus, the main objectives of this work can be introduced as follows:

1. To test the DK, DKM shell element classes in the context of FELA, and to compare them with the strict upper-bound shell elements used in literature.
2. To test the DK, DKM shell element classes in the context of SFELA.
3. To demonstrate that, although these elements cannot guarantee a strict upper bound, discretization errors are significantly lower compared to the strict upper-bound shell elements.
4. In particular, to show that the use of quadrilateral elements in the context of FELA is beneficial.

The optimization script file was generated by the finite element solver femCalc^33^. The optimization problem was solved by the optimizer MOSEK, version 10.1.27^5^.

The paper is structured as follows: Section “Upper bound formulation of the finite-element based limit analysisfor shells” presents the kinematic upper bound formulation of FELA, the failure criteria used, and the SFELA approach. Section “Finite elements used” describes the tested shell elements. Section “Convergence tests” investigates the convergence behavior of the considered shell elements on six benchmark problems. Closing remarks are given in Section “Conclusions”.

## Upper bound formulation of the finite-element based limit analysis for shells

### Continuous formulation

The upper bound formulation of FELA is based on minimization of the internal dissipation power summed over a body domain, keeping the power of external forces equal to one. In this way we get a convex, generally non-smooth, optimization problem defined in terms of velocities in the body at the instance of the plastic collapse, which has the plastic load multiplier *λ* as the objective. We consider a linear distribution of the strain across the shell thickness $\dot{\boldsymbol{\epsilon }}(x,y,z)=\dot{\boldsymbol{\varepsilon }}(x,y)+z\dot{\boldsymbol{\kappa }}(x,y)$, where $\dot{\boldsymbol{\varepsilon }}=[\dot{\varepsilon }_x,\,\dot{\varepsilon }_y,\,\dot{\gamma }_{xy}]^{\text {T}}$ is the membrane plastic strain rate, $\dot{\boldsymbol{\kappa }}=[\dot{\kappa }_x,\,\dot{\kappa }_y,\,\dot{\kappa }_{xy}]^{\text {T}}$ is the bending plastic strain rate and $\dot{\boldsymbol{\epsilon }}=[\dot{\epsilon }_x,\,\dot{\epsilon }_y,\,\dot{\curlyvee }_{xy}]^{\text {T}}$ is the in-plane plastic strain rate at coordinate *z*. The primal upper bound formulation in its continuous form has the form

1 $$\begin{aligned} \min _{\dot{\boldsymbol{q}}}&~D_{\text {p}}(\dot{\boldsymbol{u}},\dot{\boldsymbol{\varphi }}) \end{aligned}$$

2 $$\begin{aligned} \text {s.t.}&~~ \int _{\Omega } \boldsymbol{p}^{\text {T}}\dot{\boldsymbol{u}}\,\text {d}\Omega +\int _{\Gamma _{\text {p}}} \boldsymbol{p}_{\Gamma }^{\text {T}}\dot{\boldsymbol{u}}\,\text {d}\Gamma _{\text {p}}=1, \end{aligned}$$

3 $$\begin{aligned}&~~ \dot{u_i}\Big |_{\Gamma _{\text {u},i}}=0,~i=1,2,3, \end{aligned}$$

4 $$\begin{aligned}&~~ \dot{\varphi _i}\Big |_{\Gamma _{\varphi ,i}}=0,~i=1,2,3, \end{aligned}$$

where $D_{\text {p}}$ is the dissipation power in the considered body *Ω*, $\boldsymbol{p}=[p_x,\,p_y,\,p_z]^{\text {T}}$ represents the pressure acting on *Ω*, $\boldsymbol{p}_{\Gamma }$ is the pressure vector acting on the part of the domain boundary $\Gamma _{\text {p}}\subset \partial \Omega$. The subset $\Gamma _{\text {u},i}\subset \Gamma$ denotes the part of the boundary where $\dot{u}_i=0$, for *i=1,2,3*, and $\Gamma _{\varphi ,i}\subset \Gamma$ denotes the part of the boundary where $\dot{\varphi }_i=0$, for *i=1,2,3*. In the case of the stress-resultant-based failure criteria we have

5 $$\begin{aligned} D_{\text {p}}(\dot{\boldsymbol{{\mathcal {E}}}}) =\int _{\Omega } d_{\text {p}}( \dot{\boldsymbol{{\mathcal {E}}}}(x,y))\,\,\text {d}\Omega , \end{aligned}$$

where $$\dot{\boldsymbol{{\mathcal {E}}}}(x,y)=[\begin{array}{c} \dot{\boldsymbol{\kappa }},~\dot{\boldsymbol{\varepsilon }}\end{array}]^{\text {T}}$$ and

6 $$\begin{aligned} d_{\text {p}}(\dot{\boldsymbol{{\mathcal {E}}}})&=\max _{\boldsymbol{m},\boldsymbol{n}~\text {s.t.}~f(\boldsymbol{m},\boldsymbol{n})\le 1} \boldsymbol{m}^{\text {T}}\dot{\boldsymbol{\kappa }}+\boldsymbol{n}^{\text {T}}\dot{\boldsymbol{\varepsilon }}, \end{aligned}$$

where $\boldsymbol{n}=[n_x,\,n_y,\,n_{xy}]^{\text {T}}$ is the vector of internal in-plane forces, $\boldsymbol{m}=[m_x,\,m_y,\,m_{xy}]^{\text {T}}$ is the vector of internal moments and *f* is the failure criterion. In the case of the stress-based failure criteria we have

7 $$\begin{aligned} D_{\text {p}}(\dot{\boldsymbol{\epsilon }}) =\int _{\Omega }\int _{-\frac{h}{2}}^{\frac{h}{2}}d_{\text {p}}^{z}(\dot{\boldsymbol{\epsilon }}(x,y,z))\,\,\text {d}z\,\text {d}\Omega , \end{aligned}$$

where $$\dot{\boldsymbol{\epsilon }}(x,y,z)=\dot{\boldsymbol{\varepsilon }}(x,y)+z\dot{\boldsymbol{\kappa }}(x,y)$$ and

8 $$\begin{aligned} d_{\text {p}}^z(\dot{\boldsymbol{\epsilon }})&=\max _{\boldsymbol{\sigma }~\text {s.t.}~f(\boldsymbol{\sigma })\le 1} \boldsymbol{\sigma }:\dot{\boldsymbol{\epsilon }}, \end{aligned}$$

where $\boldsymbol{\sigma }=[\sigma _x,\sigma _y,\tau _{xy}]^{\text {T}}$ is the stress vector. All failure criteria used in this article, given either by Eq. (6) or (8), have explicit forms defined only in terms of the plastic strain rates. Note, that these formulations can be easily extended to the Mindlin case, in which the failure criterion *f* also depends on transversal shear stresses^7^.

### Failure criteria used in our test problems

In the framework of FELA, which yields the second order cone optimization problem^34^, only those failure criteria can be used, that belong to the class of associated plasticity and which can simultaneously be formulated in the form of a second order cone (An *n*-dimensional second order cone is defined as $Q^n=\left\{ \boldsymbol{x}\in {\mathbb {R}}^n:~x_1\ge \sqrt{x_1^2+\dots +x_n^2}\right\}$). In our test problems, we use both stress-resultant-based and stress-based failure criteria to provide the widest range of tested scenarios.

### Stress-resultant based failure criteria

#### Approximative Ilyushin failure criterion

In 1948, A.A. Ilyushin proposed an exact parametrization of the von Mises failure surface, which, however, cannot be expressed in the form of a second-order cone^35^. A frequently used approximation of the exact Ilyushin parametrization, also proposed by A.A.Ilyushin, is given by intersection of two second order cones:

9 $$\begin{aligned} f&=\max (f_1,f_2), \end{aligned}$$

10 $$\begin{aligned} f_i&= \frac{\boldsymbol{n}^{\text {T}}\boldsymbol{P}\boldsymbol{n}}{n_{\text {p}}^2}\!+\!\frac{\boldsymbol{m}^{\text {T}}\boldsymbol{P}\boldsymbol{m}}{m_{\text {p}}^2}\!+\! \frac{(-1)^i}{\sqrt{3}}\frac{\boldsymbol{m}^{\text {T}}\boldsymbol{P}\boldsymbol{n}}{n_{\text {p}}m_{\text {p}}} \le 1,~i=1,2, \end{aligned}$$

11 $$\begin{aligned} \boldsymbol{P}&=\left[ \begin{array}{ccc} 1 & -\frac{1}{2}& 0\\ -\frac{1}{2}& 1 & 0\\ 0 & 0 & 3 \end{array} \right] , \end{aligned}$$

where $n_{\text {p}}=\sigma _0h$ is the maximum plastic in-plane force and $m_{\text {p}}=\sigma _0\frac{h^2}{4}$ is the maximum plastic moment. Note, that if one restricts to the pure in-plane situation ($\boldsymbol{m}=\boldsymbol{0}$) or to the pure bending situation ($\boldsymbol{n}=\boldsymbol{0}$), then Eqs. (9-11) coincide with the von Mises failure criterion. We decompose the matrix $\boldsymbol{P}$ as follows:

12 $$\begin{aligned} \boldsymbol{P}=\boldsymbol{R}^{\text {T}}\boldsymbol{R},~\boldsymbol{R}= \left[ \begin{array}{ccc} ~~\frac{\sqrt{3}}{2} & 0 & 0\\ -\frac{1}{2} & 1 & 0\\ 0 & 0 & \sqrt{3} \end{array}\right] . \end{aligned}$$

The final expression of the dissipation power area-density has the form^20^

13 $$\begin{aligned} d_{\text {p}}(\dot{\boldsymbol{{\mathcal {E}}}})&=\sum _{i=1}^2||\mathcal {{\tilde{R}}}_i^{-\text {T}} \dot{\boldsymbol{{\mathcal {E}}}}_i||, \end{aligned}$$

14 $$\begin{aligned} ~~\dot{\boldsymbol{{\mathcal {E}}}}&= \left[ \begin{array}{c} \boldsymbol{\dot{\kappa }} \\ \boldsymbol{\dot{\varepsilon }} \end{array} \right] =\dot{\boldsymbol{{\mathcal {E}}}}_1+\dot{\boldsymbol{{\mathcal {E}}}}_2= \left[ \begin{array}{c} \boldsymbol{\dot{\kappa }}_1 \\ \boldsymbol{\dot{\varepsilon }}_1 \end{array} \right] + \left[ \begin{array}{c} \boldsymbol{\dot{\kappa }}_2 \\ \boldsymbol{\dot{\varepsilon }}_2 \end{array} \right] , \end{aligned}$$

15 $$\begin{aligned} \mathcal {{\tilde{R}}}_{i}^{-\text {T}}&=\left[ \begin{array}{cc} \boldsymbol{0} & n_{\text {p}} \boldsymbol{R}^{-\text {T}} \\ \frac{m_{\text {p}}}{\sqrt{1-\alpha ^2}} \boldsymbol{R}^{-\text {T}} & -\frac{\alpha n_{\text {p}}}{\sqrt{1-\alpha ^2}}\boldsymbol{R}^{-\text {T}} \\ \end{array} \right] ,~\alpha =\frac{(-1)^i}{2\sqrt{3}},~i=1,2, \end{aligned}$$

16 $$\begin{aligned} ~\boldsymbol{R}^{-\text {T}}&=\left[ \begin{array}{ccc} \frac{2}{\sqrt{3}} & \frac{1}{\sqrt{3}} & 0 \\ 0& 1& 0\\ 0& 0& \frac{1}{\sqrt{3}} \\ \end{array} \right] . \end{aligned}$$

#### Von Mises failure criterion for in-plane loading

The von Mises failure criterion for the case of in-plane loading is the special case of the Ilyushin criterion for $\boldsymbol{m}=0$: $f=\frac{\boldsymbol{n}^{\text {T}}\boldsymbol{P}\boldsymbol{n}}{n_{\text {p}}^2}\le 1$. The final expression of the dissipation power area-density has the form

17 $$\begin{aligned} d_{\text {p}}(\boldsymbol{\dot{\varepsilon }})=n_{\text {p}}||\boldsymbol{R}^{-\text {T}} \boldsymbol{\dot{\varepsilon }}||. \end{aligned}$$

#### Von Mises failure criterion for pure bending

The von Mises failure criterion for pure bending is the special case of the Ilyushin criterion for $\boldsymbol{n}=0$: $f=\frac{\boldsymbol{m}^{\text {T}}\boldsymbol{P}\boldsymbol{m}}{m_{\text {p}}^2}\le 1$. The final expression of the dissipation power area-density has the form

18 $$\begin{aligned} d_{\text {p}}(\boldsymbol{\dot{\kappa }})=m_{\text {p}}||\boldsymbol{R}^{-\text {T}} \boldsymbol{\dot{\kappa }}||. \end{aligned}$$

### Stress based failure criteria

#### Plane-stress Tresca failure criterion

The Tresca in-plane plane stress failure criterion is given by

19 $$\begin{aligned} f=\frac{\max (|\sigma _2-\sigma _1|,|\sigma _1|,|\sigma _2|)}{\sigma _0}\le 1, \end{aligned}$$

where $\sigma _1, \sigma _2$ are eigenstresses. The final expression of the dissipation power density has the form

20 $$\begin{aligned} d_{\text {p}}^z(\dot{\boldsymbol{\epsilon }})&=\sigma _0\max \left[ \left| \frac{\dot{\epsilon }_x+\dot{\epsilon }_y-\sqrt{A}}{2}\right| , \left| \frac{\dot{\epsilon }_x+\dot{\epsilon }_y+\sqrt{A}}{2}\right| ,|\dot{\epsilon }_x+\dot{\epsilon }_y|\right] , \end{aligned}$$

where $A=(\dot{\epsilon }_x - \dot{\epsilon }_y)^2+\dot{\curlyvee }_{xy}^2$. For the derivation and implementation details, refer to Appendix 1.

#### Plane-strain Tsai-Wu failure criterion

The Tsai-Wu yield criterion is a single-surface, quadratic failure model used to describe materials exhibiting orthotropic behavior^36^. In the plane-strain approximation we consider $\dot{\varepsilon }_z=\dot{\gamma }_{xz}=\dot{\gamma }_{yz}=0$. This criterion is extracted from the three-dimensional Tsai-Wu criterion under the assumption of transversal isotropy ($f_{\text {t}y}\!=\!f_{\text {t}z},\,f_{\text {c}y}\!=\!f_{\text {c}z}$), under the assumption of a zero coefficient of the cross-term $\sigma _x\sigma _y$, and under the assumption of a negligible influence of transversal shear stresses:

21 $$\begin{aligned}&f=\sigma _x\left[ \frac{1}{f_{\text {t}x}}-\frac{1}{f_{\text {c}x}}\right] + (\sigma _y+\sigma _z)\left[ \frac{1}{f_{\text {t}y}}-\frac{1}{f_{\text {c}y}}\right] + \frac{\sigma _x^2}{f_{\text {t}x}f_{\text {c}x}}+\frac{\sigma _y^2+\sigma _z^2}{f_{\text {t}y}f_{\text {c}y}} + \frac{\tau _{xy}^2}{f_{\text {v}xy}^2}\le 1. \end{aligned}$$

The final expression of the dissipation power density has the form

22 $$\begin{aligned} d_{\text {p}}^z(\dot{\boldsymbol{\epsilon }})=&\frac{f_{\text {t}x}-f_{\text {c}x}}{2}\dot{\epsilon }_x+\frac{f_{\text {t}y}-f_{\text {c}y}}{2}\dot{\epsilon }_y + \sqrt{(\boldsymbol{R}^{-\text {T}}\dot{\boldsymbol{\epsilon }})^{\text {T}}\boldsymbol{R}^{-\text {T}}\dot{\boldsymbol{\epsilon }}}, \end{aligned}$$

where

23 $$\begin{aligned} \boldsymbol{R}^{-\text {T}}&=\left[ \begin{array}{ccc} \sqrt{\chi f_{\text {t}x}f_{\text {c}x}} & 0 & 0\\ 0 & \sqrt{\chi f_{\text {t}y}f_{\text {c}y}} & 0\\ 0 & 0 & \sqrt{\chi }f_{\text {v}xy}\\ \end{array}\right] , \end{aligned}$$

where $\chi =1+\frac{1}{4}\frac{(f_{\text {t}x}-f_{\text {c}x})^2}{f_{\text {t}x}f_{\text {c}x}}+\frac{1}{2}\frac{(f_{\text {t}y}-f_{\text {c}y})^2}{f_{\text {t}y}f_{\text {c}y}}$. For the derivation, refer to Appendix 1.

### Discretized formulation

We discretize at first the formulation based on the Ilyushin failure criterion, i.e. Eqs. (1-5) and (13-16). We consider shell elements that produce a continuous displacement field with six degrees of freedom per node. In what follows, we introduce the solution vector at a node as $\dot{\boldsymbol{q}}=[\dot{\varphi }_X,\, \dot{\varphi }_Y,\, \dot{\varphi }_Z,\, \dot{u}_X,\, \dot{u}_Y,\, \dot{u}_Z]^{\text {T}}$. The total vector of unknown velocities, consisting of solutions at all nodes, is denoted by $\dot{\boldsymbol{\mathfrak {q}}}$. The integral in Eq. (5) is evaluated by means of the Gauss quadrature in $N_q$ quadrature point within each element. The strain matrix in element *e* and quadrature point *q* of dimensions $6\times \#\text {dofs}$, where $\#\text {dofs}$ is the total number of kinematic dofs, is denoted by $\boldsymbol{B}_{eq}$ (the matrix $\boldsymbol{B}_{eq}$ has however at most 6*n* nonzero columns, where *n* is a number of element nodes). The corresponding strain vector of length 6 is denoted by $\dot{\boldsymbol{{\mathcal {E}}}}_{eq}$. The primal formulation, denoted by (P), in the discretized form yields

24 $$\begin{aligned} \min _{\dot{\boldsymbol{\mathfrak {q}}}}&~\,\sum _{e=1}^{N_{e}} \sum _{q=1}^{N_q} J_{eq} w_q \sum _{i=1}^2 s_{ieq}, \end{aligned}$$

25 $$\begin{aligned} \text {s.t.}&~~\sum _{i=1}^2\underbrace{\mathcal {{\tilde{R}}}_i^{\text {T}}\boldsymbol{e}_{ieq}}_{\boldsymbol{\dot{\boldsymbol{{\mathcal {E}}}}}_{ieq}}-\boldsymbol{B}_{eq}\dot{\boldsymbol{\mathfrak {q}}}=\boldsymbol{0},~~e=1,\dots ,N_{e},~q=1,\dots ,N_q, \end{aligned}$$

26 $$\begin{aligned}&~~ ||\boldsymbol{e}_{ieq}|| \le s_{ieq},~~i=1,2,~~e=1,\dots ,N_{e},~q=1,\dots ,N_q, \end{aligned}$$

27 $$\begin{aligned}&~~ \boldsymbol{\mathfrak {f}}^{\text {T}}\dot{\boldsymbol{\mathfrak {q}}}=1, \end{aligned}$$

28 $$\begin{aligned}&~~ \boldsymbol{H}\dot{\boldsymbol{\mathfrak {q}}}=0, \end{aligned}$$

where $J_{eq}$ is the Jacobian in the element *e* and in the Gauss point *q*, $w_q$ is the Gauss quadrature weight, $\boldsymbol{H}$ is the matrix prescribing zero boundary conditions, with $\mathcal {{\tilde{R}}}_i$ defined in Eq. (15). The dual formulation of the primal problem, denoted by (D), is computationally more efficient^24^:

29 $$\begin{aligned} \max _{\boldsymbol{m}}~&\beta \end{aligned}$$

30 $$\begin{aligned} \text {s.t.}&~~\mathcal {{\tilde{R}}}_i\boldsymbol{m}_{eq}+\boldsymbol{s}_{ieq}=\boldsymbol{0},~~i=1,2,~~e=1,\dots ,N_{e},~~q=1,\dots ,N_q, \end{aligned}$$

31 $$\begin{aligned}&~~||\boldsymbol{s}_{ieq}||\le s_{ieq}^*:=J_{eq}w_q,~~i=1,2,~~e=1,\dots ,N_{e},~~q=1,\dots ,N_q, \end{aligned}$$

32 $$\begin{aligned}&~~ \sum _{e=1}^{N_{e}} \sum _{q=1}^{N_q} \boldsymbol{B}^{\text {T}}_{eq} \boldsymbol{m}_{eq} - \boldsymbol{H}^{\text {T}}\boldsymbol{r} - \beta \boldsymbol{\mathfrak {f}} =\boldsymbol{0}, \end{aligned}$$

where Eq. (31) defines yield surfaces at each element, and each quadrature point, and Eq. (32) equates nodal force components at each degree of freedom (dof). The term $\boldsymbol{H}^{\text {T}}\boldsymbol{r}$ represents reaction forces in fixed dofs. If this information is not needed, the fixed dofs can be excluded from the calculation. The term $\boldsymbol{\mathfrak {f}}$ represents a vector of external nodal forces. In the case of a single yield surface, the internal force $\boldsymbol{m}_{eq}$ can be eliminated from the system (by using $\boldsymbol{m}_{eq}=-\mathcal {{\tilde{R}}}^{-1}\boldsymbol{s}_{eq}$, as follows from Eq. (30)), which further simplifies the dual formulation:

33 $$\begin{aligned} \max _{\boldsymbol{s}}~&\beta \end{aligned}$$

34 $$\begin{aligned} \text {s.t.}&~~||\boldsymbol{s}_{eq}||\le s_{eq}^{*}:=J_{eq} w_q,~~e=1,\dots ,N_{e},~~q=1,\dots ,N_q,\end{aligned}$$

35 $$\begin{aligned}&~~ \sum _{e=1}^{N_{e}} \sum _{q=1}^{N_q} \boldsymbol{B}^{\text {T}}_{eq} \mathcal {{\tilde{R}}}^{-1}\boldsymbol{s}_{eq} + \boldsymbol{H}^{\text {T}}\boldsymbol{r} + \beta \boldsymbol{\mathfrak {f}} =\boldsymbol{0}. \end{aligned}$$

The primal formulation based on the stress-based failure criterion, denoted by (PT), is discretized as follows:

36 $$\begin{aligned} \min _{\dot{\boldsymbol{\mathfrak {q}}}}&~\,\frac{h}{2}\sum _{e=1}^{N_{e}} \sum _{q=1}^{N_q} \sum _{r=1}^{N_r} J_{eq} w_q w_r d_{\text {p}}^{z}(\dot{\boldsymbol{\epsilon }}_{eqr}) , \end{aligned}$$

37 $$\begin{aligned} \text {s.t.}&~~\dot{\boldsymbol{\epsilon }}_{eqr}=\dot{\boldsymbol{\varepsilon }}_{eq}+z_r\dot{\boldsymbol{\kappa }}_{eq},~~e=1,\dots ,N_{e},~~q=1,\dots ,N_q,~~r=1,\dots ,N_r,\end{aligned}$$

38 $$\begin{aligned}&~~\dot{\boldsymbol{{\mathcal {E}}}}_{eq}=\boldsymbol{B}_{eq}\dot{\boldsymbol{\mathfrak {q}}},~~e=1,\dots ,N_q,~q=1,\dots ,N_q, \end{aligned}$$

39 $$\begin{aligned}&~~ \boldsymbol{\mathfrak {f}}^{\text {T}}\dot{\boldsymbol{\mathfrak {q}}}=1, \end{aligned}$$

40 $$\begin{aligned}&~~ \boldsymbol{H}\dot{\boldsymbol{\mathfrak {q}}}=0, \end{aligned}$$

where $w_r$ is the Gauss quadrature weight for integration across the shell thickness and $\frac{h}{2}$ is the corresponding Jacobian. Some practical tips for optimization with MOSEK are provided in Appendix 2.

### Strict upper-bound versus approximative upper bound

All known strict upper bound plate or shell elements^14,20,25^ are triangular elements with straight sides, which guarantee a constant Jacobian. This is an important property in proving that the flow rule is fulfilled at each point of the element. Quadrangle shell elements, on the other hand, equipped with additional numerical instruments like bubble functions, are known for their numerical efficiency compared to triangular elements. If the strict upper bound of the formulation is not guaranteed, then the flow rule is not fulfilled point-wise but only on average for each element (The first-order optimality condition of the maximization (6) or (8) immediately yields the flow rule. E.g., in the case of Eq.(8) we get the Lagrangian ${\mathcal {L}}\!=\!\boldsymbol{\sigma }\!:\!\dot{\boldsymbol{\varepsilon }}\!-\!\lambda (f(\boldsymbol{\sigma })\!-\!1)$, and the first order optimality condition yields $\frac{\partial {\mathcal {L}}}{\partial \boldsymbol{\sigma }}\!=\!\dot{\boldsymbol{\varepsilon }}\!-\!\lambda \frac{\partial f(\boldsymbol{\sigma })}{\partial \boldsymbol{\sigma }}\!=\!0$). In this article, we compare different shell elements that have no bounding effect together with the strict upper bound shell elements.

### Sequential finite-element-based limit analysis (SFELA)

**Fig. 1:**
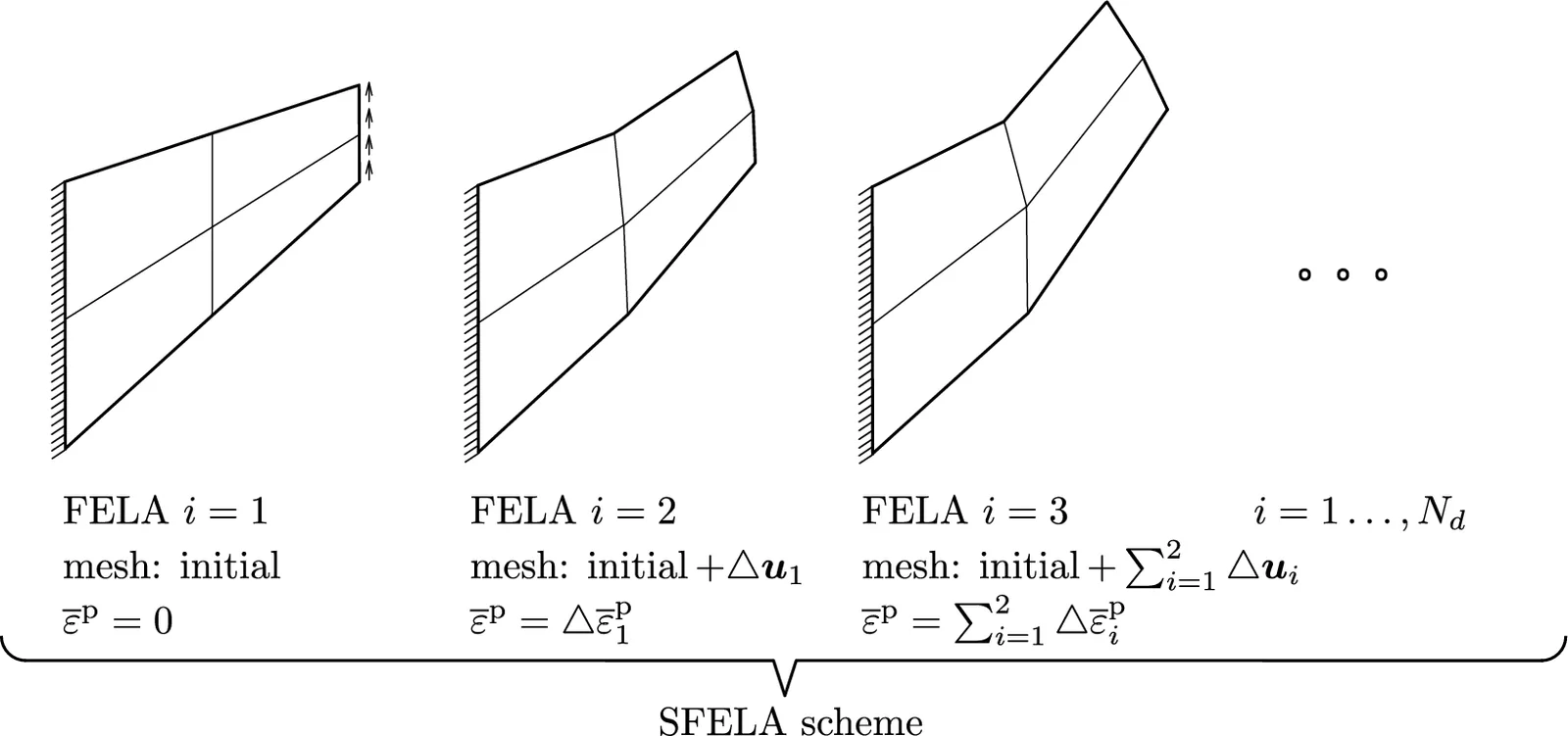
Each iteration of the SFELA scheme corresponds to a FELA simulation. The output of each FELA simulation includes the velocity $\dot{\boldsymbol{u}}_i$ and the plastic strain rate $\dot{\boldsymbol{\varepsilon }}^{\text {p}}_i$, $i=1,\dots ,N_{d}$. The mesh node positions are updated by amount $\triangle \boldsymbol{u}_i=\triangle t \dot{\boldsymbol{u}}_i,~~i=1,\dots ,N_{d}$. The accumulated plastic strain $\overline{\varepsilon }^{\text {p}}$ is then computed, and the yield strength $\sigma _0(\overline{\varepsilon }^{\text {p}})$ is updated at each step and at each Gauss point according to the strain hardening diagram.

Two basic assumptions of FELA (the assumption of small deformations and the assumption of ideal plasticity) are both restrictive for real engineering applications. The SFELA method is a modification of FELA method, first introduced by Yang^8^, which overcomes, to some extent, both of these problems. The idea of the method is based on the time integration of nodal velocities

41 $$\begin{aligned} \triangle \boldsymbol{u}_i=\triangle t \dot{\boldsymbol{u}}_i,~~i=1,\dots ,N_{d}, \end{aligned}$$

for some time step *△ t*, where $N_{d}$ is a number of nodes. In this way, we get a new deformed mesh on which FELA can be applied again to obtain the new velocity field. By repeating this procedure, we get the time-dependent displacement field of the plastic collapse. By updating the yield stress at each time step according to the current value of the accumulated plastic strain, hardening or even softening can be incorporated into the calculation, see Fig. 1. For further details, see^8,9,37^.

## Finite elements used

**Fig. 2:**
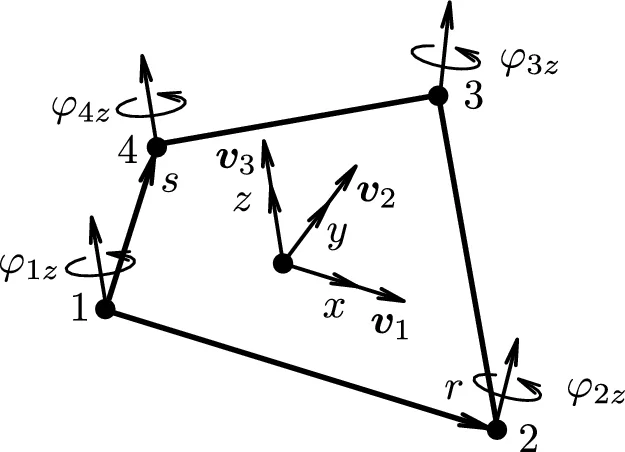
Drilling rotations and the orthonormal coordinate system $\boldsymbol{v}_1,\boldsymbol{v}_2,\boldsymbol{v}_3$ in the case of quadrangle shell element.

We use shell elements that produce a continuous displacement field used for the standard FEM analysis, which have six degrees of freedom per node (three displacements and three rotations). All used elements are listed in Table 2. The triangular plate elements (DKT, Specht, DKMT) must be equipped with a membrane element (OPT) to function as shells. Analogously, the quad plate elements (DKQ, DKMQ) must be equipped with a membrane element (OPQ) to function as shells. The DKQ and DKMQ elements only work in the planar case, so they must be further equipped with the warping matrix (the rigid links correction) to also work in the non-planar case^38^. Regarding the numerical integration of the stiffness matrix, we use 3-point quadrature for triangles and 4-point quadrature for quadrangles. In order to stabilize drilling rotations, the following penalty term is added to the minimized objective function $D_p$:

42 $$\begin{aligned}&C_{\text {d}}h\sigma _0 \sum _{i=1}^{N_{\text {e}}} \int _{\text {e}} \left| \psi (r,s)-\varphi _z(r,s)\right| \,\text {d}A, \end{aligned}$$

where

43 $$\begin{aligned} \psi (r,s)&=\frac{1}{2}\left( \frac{\partial u_y}{\partial x}(r,s)-\frac{\partial u_x}{\partial y}(r,s)\right) , \end{aligned}$$

44 $$\begin{aligned} \frac{\partial u_y}{\partial x}(r,s)&=\sum _{i=1}^n a_{i,x}(r,s)\, (\boldsymbol{v}_2(r,s))^{\text {T}} \left[ \begin{array}{c}u_{iX}\\ u_{iY}\\ u_{iZ}\end{array}\right] , \end{aligned}$$

45 $$\begin{aligned} \frac{\partial u_x}{\partial y}(r,s)&=\sum _{i=1}^n a_{i,y}(r,s)\, (\boldsymbol{v}_1(r,s))^{\text {T}} \left[ \begin{array}{c}u_{iX}\\ u_{iY}\\ u_{iZ}\end{array}\right] ,\end{aligned}$$

46 $$\begin{aligned} a_{i,x}(r,s)&=a_{i,r}(r,s)(\boldsymbol{X}^r(r,s))^{\text {T}}\boldsymbol{v}_1(r,s)+a_{i,s}(r,s)(\boldsymbol{X}^s(r,s))^{\text {T}}\boldsymbol{v}_1(r,s),\end{aligned}$$

47 $$\begin{aligned} a_{i,y}(r,s)&=a_{i,r}(r,s)(\boldsymbol{X}^r(r,s))^{\text {T}}\boldsymbol{v}_2(r,s)+a_{i,s}(r,s)(\boldsymbol{X}^s(r,s))^{\text {T}}\boldsymbol{v}_2(r,s),\end{aligned}$$

48 $$\begin{aligned} \varphi _z(r,s)&=\sum _{i=1}^n a_{i}(r,s)\, (\boldsymbol{v}_3(r,s))^{\text {T}} \left[ \begin{array}{c}\varphi _{iX}\\ \varphi _{iY}\\ \varphi _{iZ}\end{array}\right] , \end{aligned}$$

where *r,s ∈ [-1,1]* are coordinates in the element reference coordinate system, $a_i,i=1,\dots ,n$ are basis functions, $\boldsymbol{v}_1(r,s),\boldsymbol{v}_2(r,s),\boldsymbol{v}_3(r,s)$ is the local orthonormal basis at point (*r*, *s*), *n=3* for triangle elements, *n=4* for quadrangle elements, and $\varphi _z(r,s)$ is the rotation about the local normal vector at point (*r*, *s*) (see Fig. 2, for details refer to^39^). The penalty constant $C_{\text {d}}$ is set to 0.001 in all test problems, except for test problem 2 (Cook’s membrane), where the value 0.0001 is used, and except for test problem 5 (block with asymmetric holes), where a higher value of 0.1 is employed (Our numerical experience indicates that in some test problems (specifically, problems 2, 4, and 6) a smaller value of $C_{\text {d}}$ is needed. However, in other test problems (specifically, problems 3 and 5), a higher value of $C_{\text {d}}$ is required to achieve accurate outcomes). $\sigma _0$ denotes the highest plastic strength of the considered problem. For evaluation of Eq. (42) 1-point Gauss quadrature is used.

As already mentioned, FELA neglects elasticity. However, the formulation of the Reisner-Mindlin DKM elements (DKMT, DKMQ, DKMQ24, DKMQ24+) contains elastic parameters in two places: 1. Poisson’s ratio is used in the shear approximation, 2. in the case of the DKMQ24+ element, the elastic membrane stiffness matrix is used for static condensation. The actual setting of the elastic parameters is taken as follows: in the isotropic case, $E=\sigma _0,~\nu =0.5$, and in the case of the Tsai-Wu plane strain orthotropic criterion, $\nu _x=\nu _y=0,~E_x=\sqrt{\chi f_{\text {t}x} f_{\text {c}x}},~E_y=\sqrt{\chi f_{\text {t}y} f_{\text {c}y}},~G_{xy}=f_{\text {v}xy}\sqrt{\chi }$, where $\chi =1+\frac{1}{4}\frac{( f_{\text {t} x}-f_{\text {c} x})^2}{ f_{\text {t} x} f_{\text {c} x}}+\frac{1}{2}\frac{(f_{\text {t} y}-f_{\text {c} y})^2}{ f_{\text {t} y} f_{\text {c} y}}$.

## Convergence tests

All the test problems presented in this section are listed in Table 1, while all finite elements used in this article are listed in Table 2. The standard method for evaluating convergence behavior is to depict the relative error with respect to a number of dofs. A number of dofs approximately reflects the computational complexity of the problem, which allows us to compare elements of different orders. However, this can be partially misleading because optimization time also depends on a number of auxiliary variables (for example, whether the strain rate is eliminated from the formulation or not, and on the number of Gauss points used, which varies for different elements). Therefore, we also provide diagrams of the relative error with respect to the actual optimization time. It is important to note that this optimization time refers to the time needed by the MOSEK optimizer and does not include the time required for generating the script file. A computer equipped with an i7-10875H CPU and 128 GB of RAM was used for all calculations except for the one reported in Table 22, which was performed on a computer with an i7-14700HX CPU and 192 GB of RAM.

**Table 1:** Test problems.

No.	Test problem	Element type	Material model	Analysis	Formulation	Ref.
1.	Clamped plate	Plate	von Mises	FELA	(D)	^7,18,24^
2.	Cook’s membrane	Membrane	von Mises (plane stress)	FELA	(P)	^40^
3.	Simply support. spherical cap	Shell	Tresca	FELA	(PT)	^20^
4.	Clamped cylindrical tube	Shell	Approximative Ilyushin	FELA	(PT)	^20^
5.	Block with asymmetric holes	Membrane	Tsai-Wu (plane strain)	FELA	(P)	^13,16,41^
6.	Simply supported plate	Shell	Approximative Ilyushin	SFELA	(P)	^30^

**Table 2:** Used finite elements.

Element notation	Element shape	Element type	#quad- rature points	Transv. shear approx.	Implementation	Ref.	Comment
Ciria	Triangle	Membrane	1	—	From Reference	^40^	Strict upper-bound; continuous 3-node triangle
Hodge/Belytschko	Triangle	Plate	1	Kirchhoff	From Reference	^42^	Strict upper-bound; continuous 6-node triangle
Makrodimopoulos	Triangle	Plate		Kirchhoff	From Reference	^25^	Strict upper-bound; continuous 6/10/15-node triangle using Bernstein polyn. of order 2/3/4
T6	Triangle	Plate	1	Kirchhoff	From Reference	^18^	Strict upper-bound; continuous 6-node triangle
T6b	Triangle	Plate	1	Kirchhoff	From Reference	^18^	6-node triangle with a cubic bubble function
TRIC	Triangle	Shell	3	Mindlin	From Reference	^43^	=TRIangular Composite
Specht	Triangle	Plate	3	Kirchhoff	Our implementation	^44^	
DKT	Triangle	Plate	3	Kirchhoff	Our implementation	^45^	=Discrete Kirchhoff Triangle
DKQ	Quad	Plate	3	Kirchhoff	Our implementation	^46^	=Discrete Kirchhoff Quadrangle, uses the warping matrix^38^
OPT	Triangle	Membrane	3	–	Our implementation	^47^	=OPtimal Triangle
OPQ	Quad	Membrane	4	–	Our implementation	^48^	=OPtimal Quadrangle
DKMT	Triangle	Plate	3	Mindlin	Our implementation	^49^	=Discrete Kirchhoff–Mindlin Triangle
DKMQ	Quad	Plate	3	Mindlin	Our implementation	^50^	=Discrete Kirchhoff–Mindlin Quadrangle, uses the warping matrix^38^
DKMQ24	Quad	Shell	4	Mindlin	Our implementation	^51^	
DKMQ24+	Quad	Shell	4	Mindlin	Our implementation	^39^	DKMQ24$\vphantom{0}_2$+ uses the *2× 2* Gauss quadrature, without nodal moment corrections

All elements in our implementation were implemented in the program femCalc^33^ and tested on linear elastic benchmarks. Only first four elements guarantee the strict upper-bound formulation.

### Clamped pressure loaded square plate

**Fig. 3:**
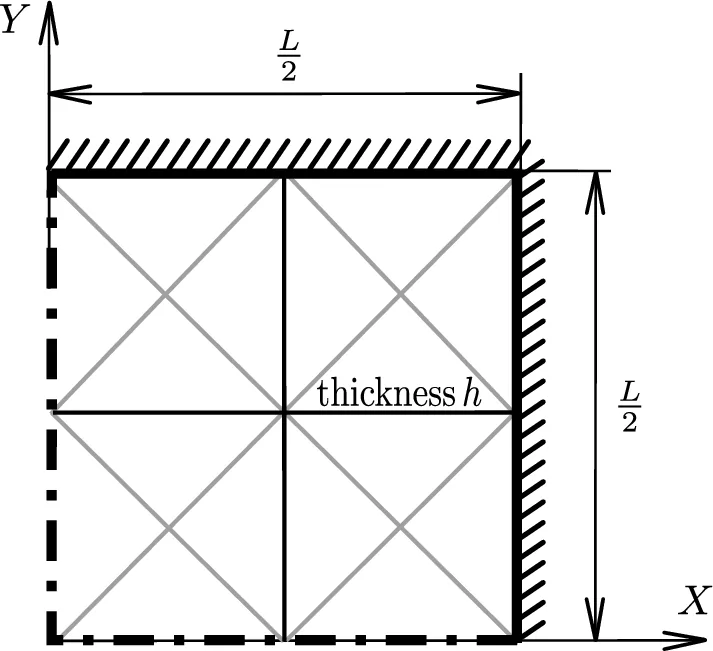
A quarter of the clamped pressure loaded square plate with a quad mesh of *2× 2*, a triangular mesh is depicted in gray. Parameters: $L=10\,\text {m},\,h=0.001\,\text {m},\,f_{\text {y}}=4\,\text {MPa}$, loading pressure $\,p=1\,\text {Pa}$.

**Fig. 4:**
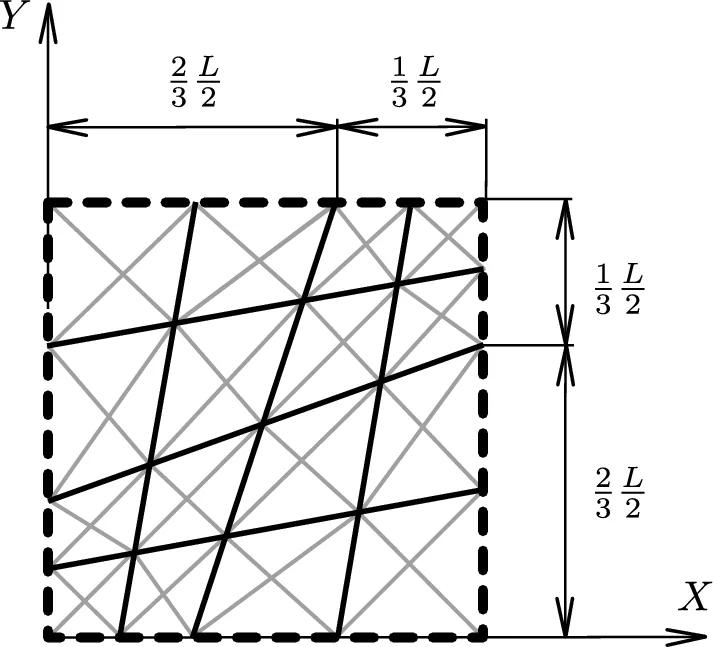
A quarter of the clamped square plate with a distorted quad mesh of *4× 4*. A triangular mesh is depicted in gray.

**Fig. 5:**
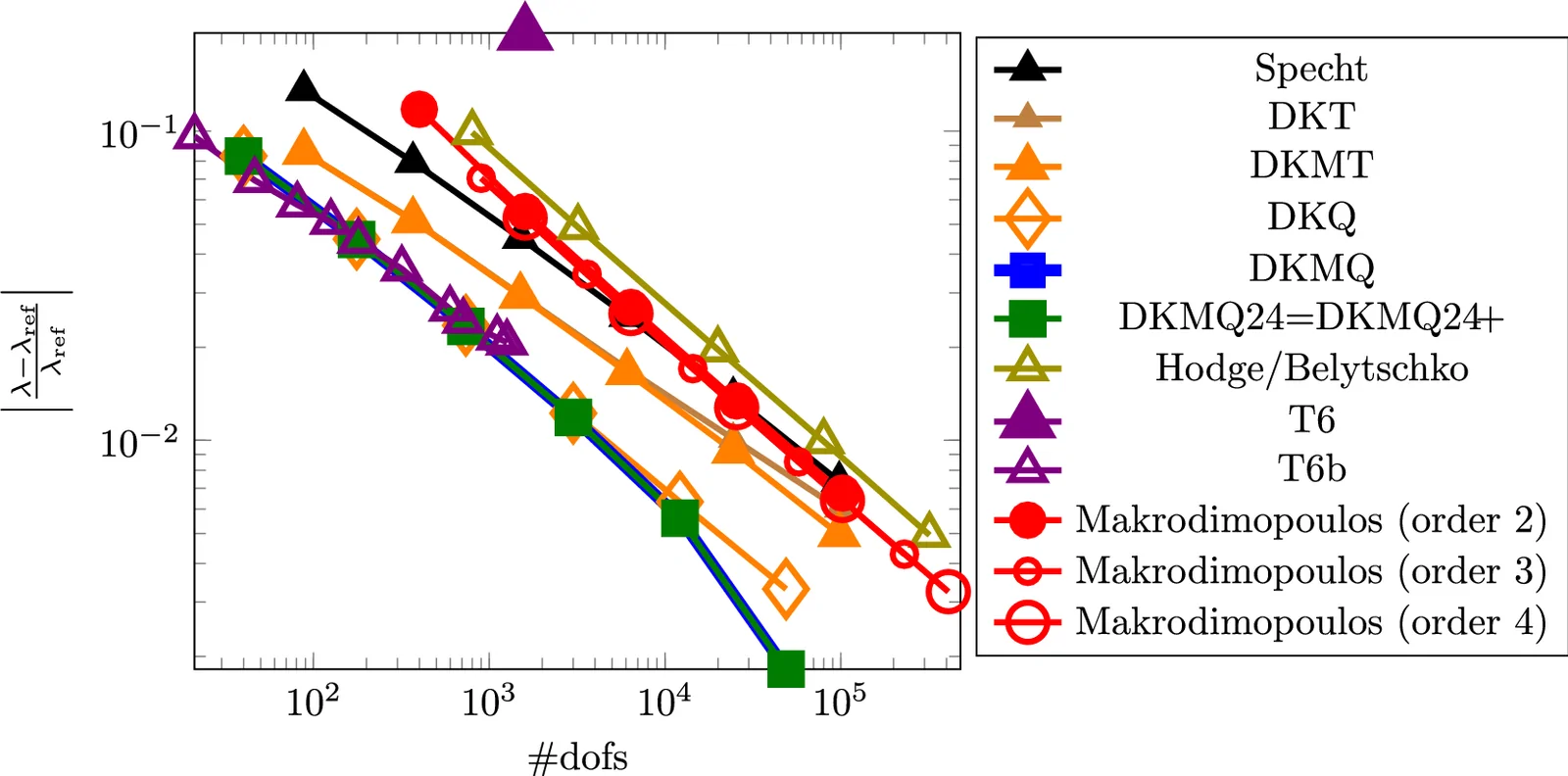
Clamped pressure loaded square plate, uniform mesh. T6b element: results are given in^18^ in terms of total optimization unknowns $N_{\text {opt}}$. The recalculation to $\#\text {dofs}$ is done as follows: $N_{\text {opt}}=\#\text {dofs}+12N_{e}+6N_{\text {edge}}$^18^, the number of edges $N_{\text {edge}}=1.5N_{e}\implies \#\text {dofs}=N-21N_{e}$. For the mesh used in^18^, we get $\#\text {dofs}=3N_{e}$, also finally we obtain $\#\text {dofs}=\frac{N_{\text {opt}}}{8}$. Hodge/Belytschko element: results are given in^24^ in terms of element number $N_{e}$, for the mesh used in^24^ we obtain $\#\text {dofs}=2N_{e}$. Makrodimopoulos elements^25^: results are valid for mesh B^25^ for which $\#\text {dofs}=(o N_{\text {s}})^2$, where element order *o=2,3,4* and $N_{\text {s}}$ is the number of elements per plate side.

**Fig. 6:**
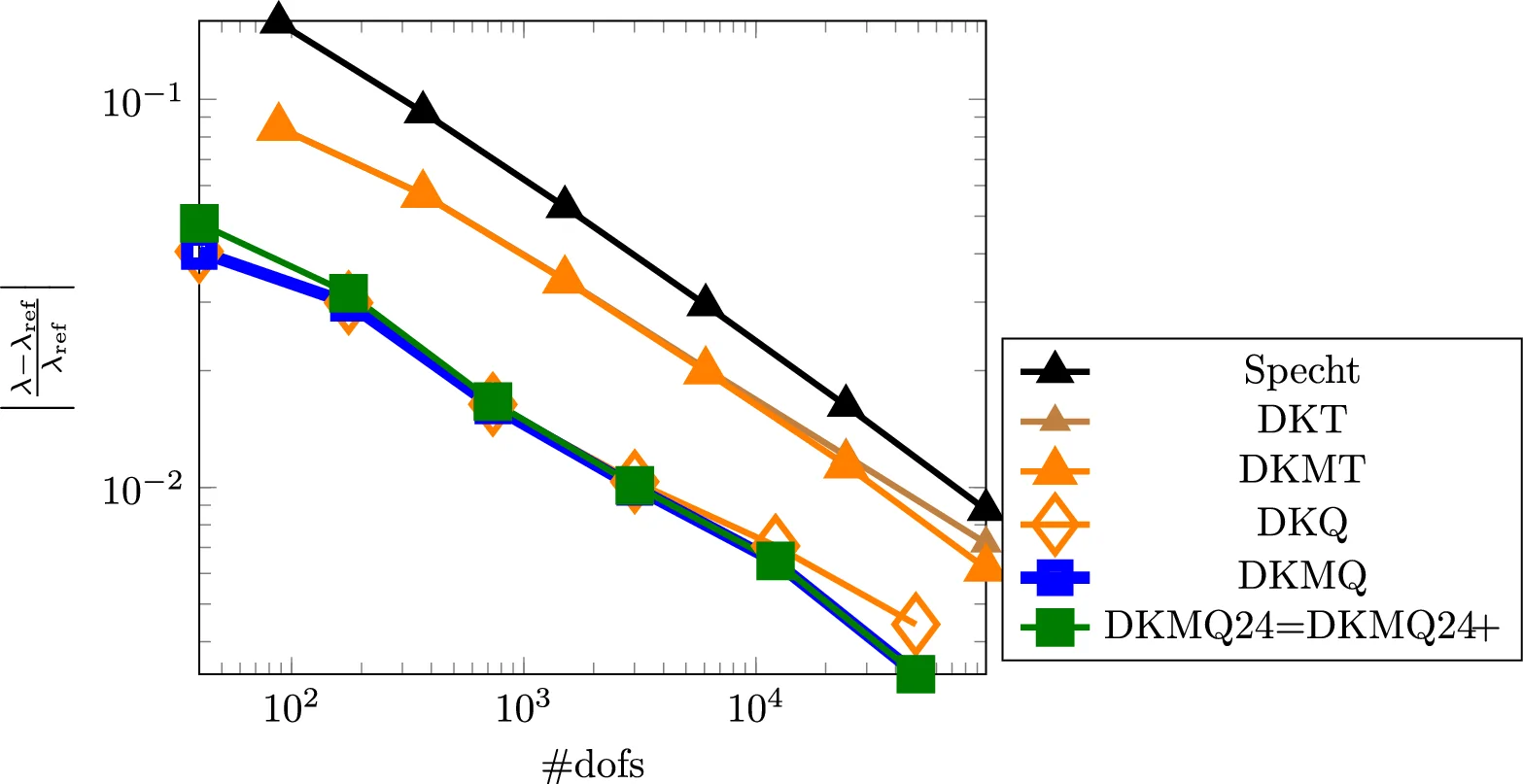
Clamped pressure loaded square plate, distorted mesh.

**Fig. 7:**
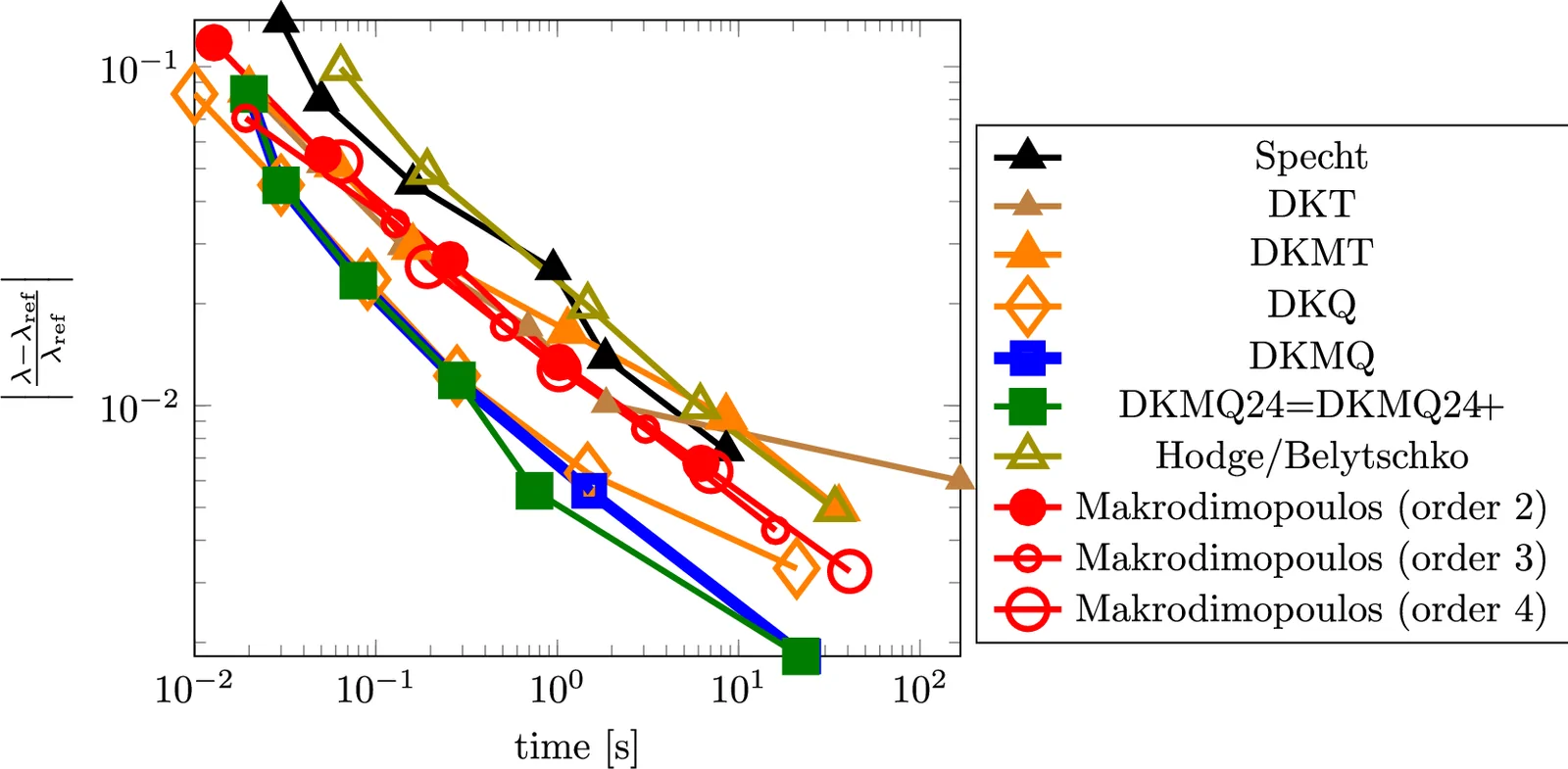
Clamped pressure loaded square plate, uniform mesh. Time results for the Hodge/Belytschko and Makrodimopoulos were calculated on the i7-2670QM, which is according to the SuperPI-32M test *0.64×*slower then i7-10875H, the time values were therefore decreased accordingly. The results for Makrodimopoulos are valid for mesh B^25^. Note that difference between Hodge/Belytschko element and Macrodimopoulos element for $o\!=\!2$, which are the same elements, is caused by different mesh types.

**Fig. 8:**
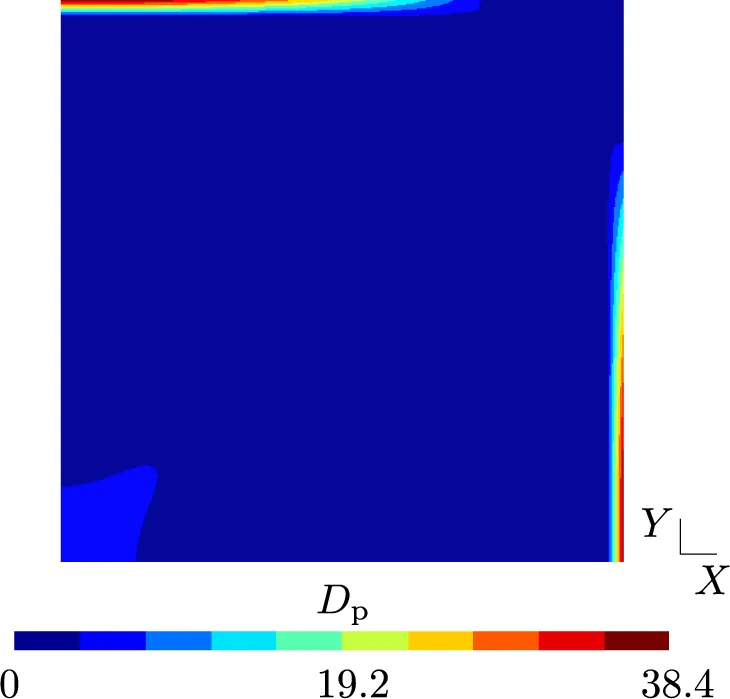
Clamped pressure loaded square plate—dissipation power at collapse.

**Fig. 9:**
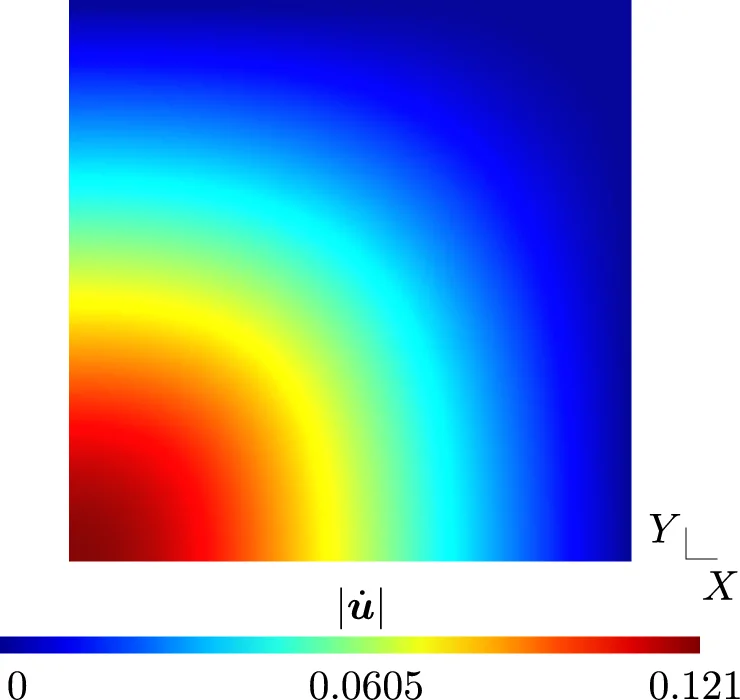
Clamped pressure loaded square plate—absolute value of velocity at collapse.

A fully clamped, pressure loaded square plate is considered (see Fig. 3 for the uniform mesh and Fig. 4 for the distorted mesh). This is a standard benchmark for limit analysis^7,18,24^. Owing to symmetry, only one quarter of the plate is analyzed. The symmetry and boundary conditions are as follows: $u_X=\varphi _Y=\varphi _Z=0$ at *X=0*, $u_Y=\varphi _X=\varphi _Z=0$ at *Y=0* and $u_X=u_Y=u_Z=\varphi _X=\varphi _Y=\varphi _Z=0$ at $X=\frac{L}{2}$ or $Y=\frac{L}{2}$. The von Mises plasticity model is used, see Eq. (18) for the corresponding dissipation power density. The numerical results for the case of the uniform mesh are summarized in Tables 3, 4, and 5 and in Fig. 5. The numerical results for the case of the distorted mesh are summarized in Tables 6 and 7 and in Fig. 6. Let us discuss the results depicted in Fig. 5. The lowest numerical error is achieved by all quadrangle elements (DKQ, DKMQ, DKMQ24, DKMQ24+) and by the T6b triangle element, which, has a slightly lower convergence order (1.000 vs. 1.056, see Table 23). The results of the strict upper-bound plate elements, i.e. T6 and the Hodge/Belytschko element exhibit higher numerical error if meassured with respect to a number of kinematic dofs than all other tested elements. When we compare two different implementations of the Kirchhoff plate elements, i.e., Specht and DKT, the Specht element exhibits the larger error. The behavior of the tested elements on the distorted mesh shown in Fig. 4 is also explored. All the tested elements converge monotonically from above to the reference solution on both uniform and distorted meshes. When we focus on the time-dependent results in Fig. 7, we see that the fastest elements are quadrangles DKQ, DKMQ, and DKMQ24=DKMQ24+ (The elements DKMQ24 and DKMQ24+ differ only in drilling rotations, which are absent in this example; hence, they produce identical results). The dissipation power and the velocity field at the instance of collapse are depicted in Figs. 8 and 9 respectively.

**Table 3:** Clamped plate, uniform mesh, dependence of the plate thickness *h* going to 0. *—a loss of convergence.

*h*	$f_{\text {t}}$	#dofs	DKMT	DKMT	#dofs	DKMQ,DKMQ24,DKMQ24+	
			*K=1*	$K\!=\!1\!\!+\!\!10\frac{h^2}{A}$		*K=1*	$K\!=\!1\!\!+\!\!10\frac{h^2}{A}$
$\text {[m]}$	[MPa]		*λ* [-]	*λ* [-]		*λ* [-]	*λ* [-]
0.1	0.04	6080	43.737	45.450	3008	42.982	45.008
		24448	42.098*	44.793	12160	41.074*	44.600
		98048	38.845*	44.462	48896	37.821*	44.370
0.01	4	6080	44.855	45.007	3008	44.646	44.679
		24448	44.529	44.734	12160	44.369	44.432
		98084	44.339	44.461	48896	44.202	44.309
0.001	400	6080	44.873	44.874	3008	44.662	44.662
		24448	44.567	44.571	12160	44.401	44.401
		98084	44.385	44.392	48896	44.266	44.269
0.0001	40000	6080	44.873	44.873	3008	44.662	44.662
		24448	44.567	44.569	12160	44.401	44.401
		98084	44.385	44.387	48896	44.266	44.268
0.00001	4000000	6080	44.873	44.873	3008	44.662	44.662
		24448	44.567	44.569	12160	44.401	44.401
		98084	44.385	44.388	48896	44.266	44.268

**Table 4:** Clamped plate (triangle elements), uniform mesh.

#elements	#dofs	Specht	DKT	DKMT
		*λ* [-]	*λ* [-]	*λ* [-]
$4\!\!\times \!\!4\!\!\times \!\!4$	88	50.114	47.900	47.897
$8\!\!\times \!\!8\!\!\times \!\!4$	368	47.631	46.397	46.392
$16\!\!\times \!\!16\!\!\times \!\!4$	1504	46.114	45.425	45.417
$32\!\!\times \!\!32\!\!\times \!\!4$	6080	45.235	44.873	44.855
$64\!\!\times \!\!64\!\!\times \!\!4$	24448	44.731	44.568	44.530
$128\!\!\times \!\!128\!\!\times \!\!4$	98048	44.447	44.387	44.341

Reference solution: $$\lambda _{\text {ref}}=44.122$$ (^25^).

**Table 5:** Clamped plate (quad elements), uniform mesh.

#elements	#dofs	DKQ	DKMQ	DKMQ24	DKMQ24+
		*λ* [-]	*λ* [-]	*λ* [-]	*λ* [-]
$4\!\!\times \!\!4$	40	47.788	47.786	47.786	47.786
$8\!\!\times \!\!8$	176	46.097	46.092	46.092	46.092
$16\!\!\times \!\!16$	736	45.161	45.153	45.153	45.153
$32\!\!\times \!\!32$	3008	44.662	44.646	44.646	44.646
$64\!\!\times \!\!64$	12160	44.401	44.369	44.369	44.369
$128\!\!\times \!\!128$	48896	44.268	44.202	44.202	44.202

Reference solution: $$\lambda _{\text {ref}}=44.122$$ (^25^).

**Table 6:** Clamped plate (triangle elements), distorted mesh.

#elements	#dofs	Specht	DKT	DKMT
		*λ* [-]	*λ* [-]	*λ* [-]
$4\!\!\times \!\!4\!\!\times \!\!4$	88	51.093	47.858	47.856
$8\!\!\times \!\!8\!\!\times \!\!4$	368	48.213	46.634	46.630
$16\!\!\times \!\!16\!\!\times \!\!4$	1504	46.455	45.637	45.630
$32\!\!\times \!\!32\!\!\times \!\!4$	6080	45.425	45.014	45.000
$64\!\!\times \!\!64\!\!\times \!\!4$	24448	44.839	44.654	44.625
$128\!\!\times \!\!128\!\!\times \!\!4$	98048	44.508	44.439	44.395

Reference solution: $$\lambda _{\text {ref}}=44.122$$ (^25^).

**Table 7:** Clamped plate (quad elements), distorted mesh.

#elements	#dofs	DKQ	DKMQ	DKMQ24	DKMQ24+
		*λ* [-]	*λ* [-]	*λ* [-]	*λ* [-]
$4\!\!\times \!\!4$	40	45.911	45.909	46.236	46.236
$8\!\!\times \!\!8$	176	45.442	45.438	45.518	45.518
$16\!\!\times \!\!16$	736	44.844	44.837	44.856	44.856
$32\!\!\times \!\!32$	3008	44.578	44.563	44.568	44.568
$64\!\!\times \!\!64$	12160	44.433	44.406	44.407	44.407
$128\!\!\times \!\!128$	48896	44.318	44.268	44.268	44.268

Reference solution: $$\lambda _{\text {ref}}=44.122$$ (^25^).

We checked for the presence of shear locking in the case of the Mindlin elements of the DKM family (DKMT, DKMQ, DKMQ24, DKMQ24+). When the plate thickness *h* is limited to zero (increasing at the same time plastic strengths, so that the resulting load factor *λ* keeps the same order), the load factor converges to a fixed value, i.e., no presence of shear locking is observed, see Table 3. However, we have encountered numerical problems in the case of moderate plate thickness, where the resulting plastic multiplier loses converge with respect to the number of degrees of freedom on finer meshes (see lines with marked with a star (*) in Table 3) (in our case only for $L=0.1\,\text {m}$). This problem occurs only in this example and only for clamped boundary conditions. In the limit the plastic load multiplier goes to zero (Possibly, the condition number of the resulting optimization problem worsens in this case, and the SOCP optimizer is unable to find the correct minimizer). The FE formulation of all these four shell elements is based on the following interpolation formula^39,49,50^:

49 $$\begin{aligned} \boldsymbol{B}_{\text {b}} = \boldsymbol{B}_{\text {b}\varphi }+\boldsymbol{B}_{\text {b}\triangle \beta }\boldsymbol{A}_n. \end{aligned}$$

We found out that if the formula is adopted as follows:

50 $$\begin{aligned} \boldsymbol{B}_{\text {b}} = \boldsymbol{B}_{\text {b}\varphi }+K\boldsymbol{B}_{\text {b}\triangle \beta }\boldsymbol{A}_n, \end{aligned}$$

where *K>1* is to be understood as a numerical parameter, then convergence is recovered for sufficiently high values of *K* for any plate thickness *h*. We can also set a higher *K* for smaller thicknesses and finer meshes, i.e., for example, as $K\!=\!1\!+\!10\frac{h^2}{A}$, see Table 3. However, the interpretation of this modification remains unclear.

In conclusion, the DKMT, DKMQ, DKMQ24, DKMQ24+ elements always work well for the small shell thickness (in comparison to the shell characteristic length), but for moderate thickness, convergence with respect to the number of degrees of freedom should always be checked. We also note that the tested Kirchhoff elements (DKT, Specht, DKQ) do not exhibit this problem.

### Cook’s membrane in plane stress

**Fig. 10:**
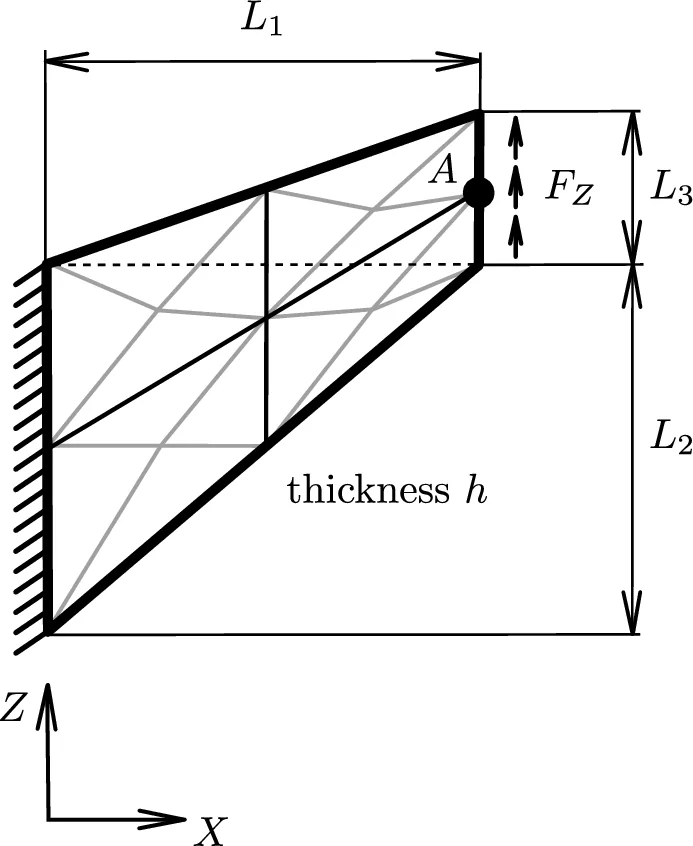
Cook’s membrane with mesh *2× 2*. Parameters: $L_1=4.8\,\text {m},\,L_2=4.4\,\text {m},\,L_3=1.6\,\text {m},\,h=1\,\text {m}$, yield strength $\sigma _0=\sqrt{3}\,\text {Pa}$, loading force $F_Z=1\,\text {N}$, test point *A*.

**Fig. 11:**
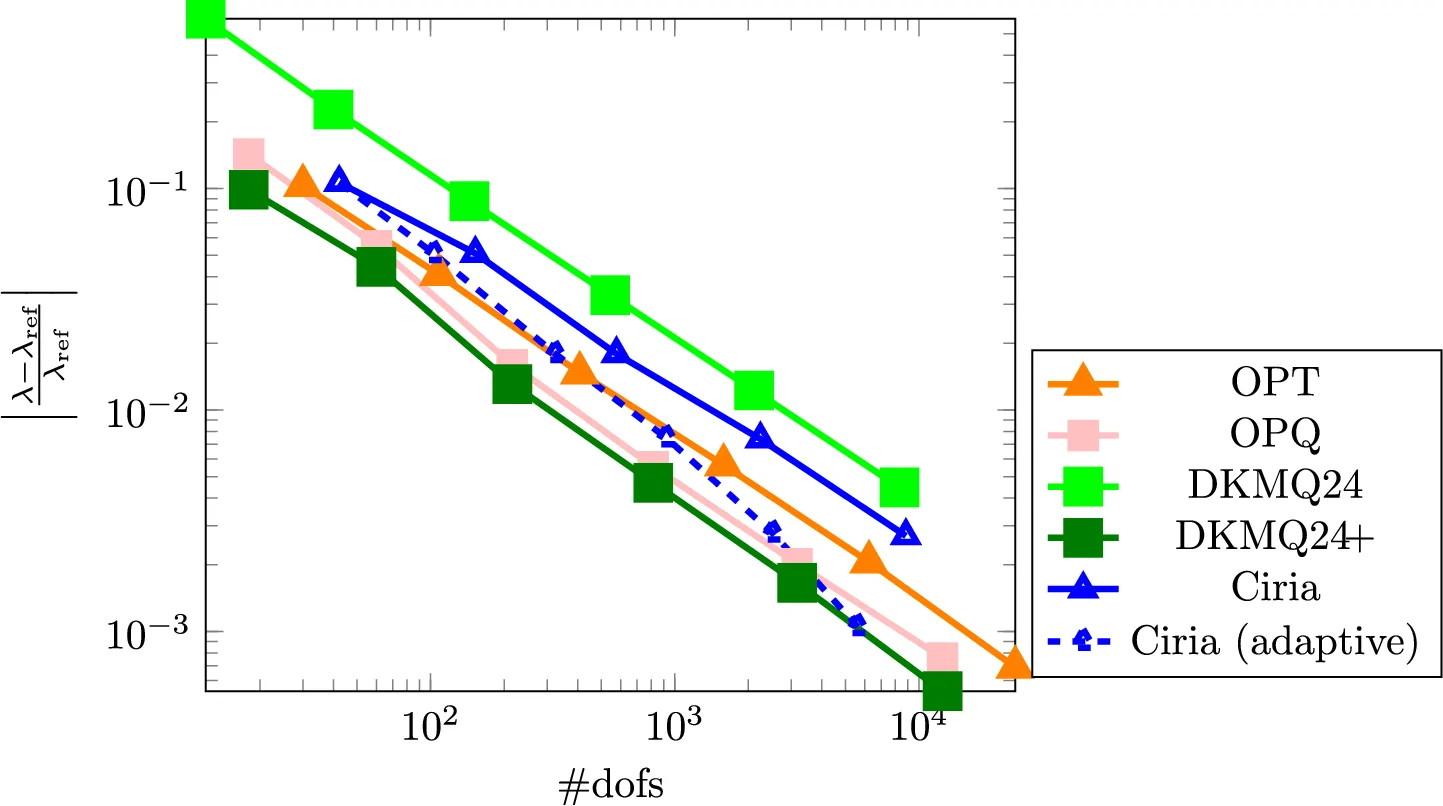
Cook’s membrane. The results for the Ciria element are given in^40^ in terms of the number of elements $N_{e}$. $\#\text {dofs}$ is approximated in this case by the formula valid for the uniform mesh $\#\text {dofs}=N_{e}+\sqrt{2N_{e}}$.

**Fig. 12:**
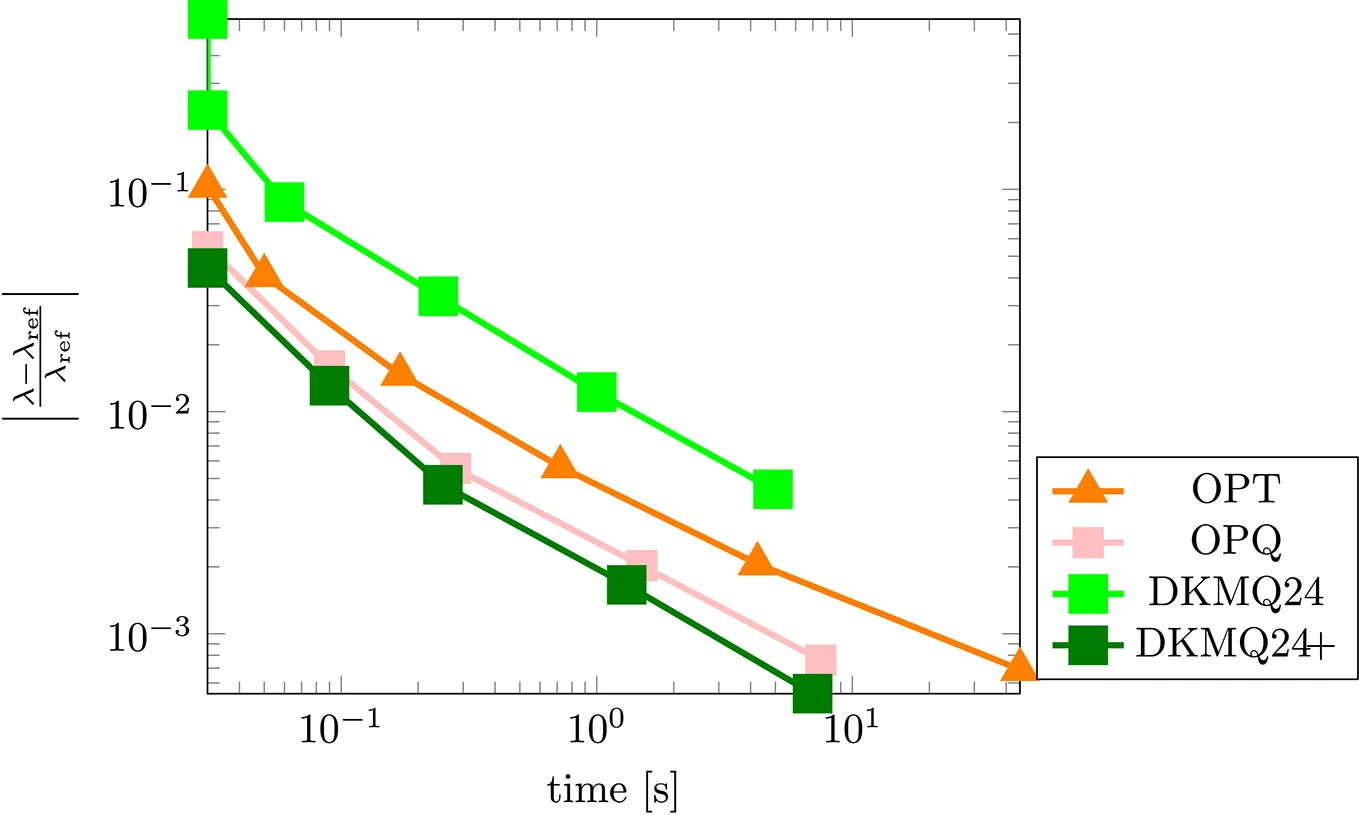
Cook’s membrane.

**Fig. 13:**
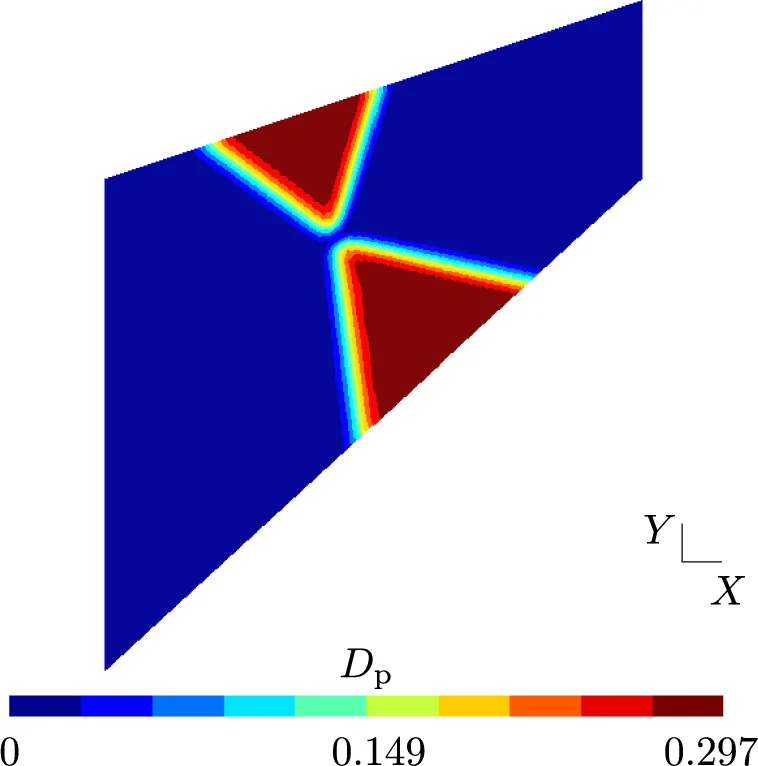
Cook’s membrane—dissipation power at collapse.

**Fig. 14:**
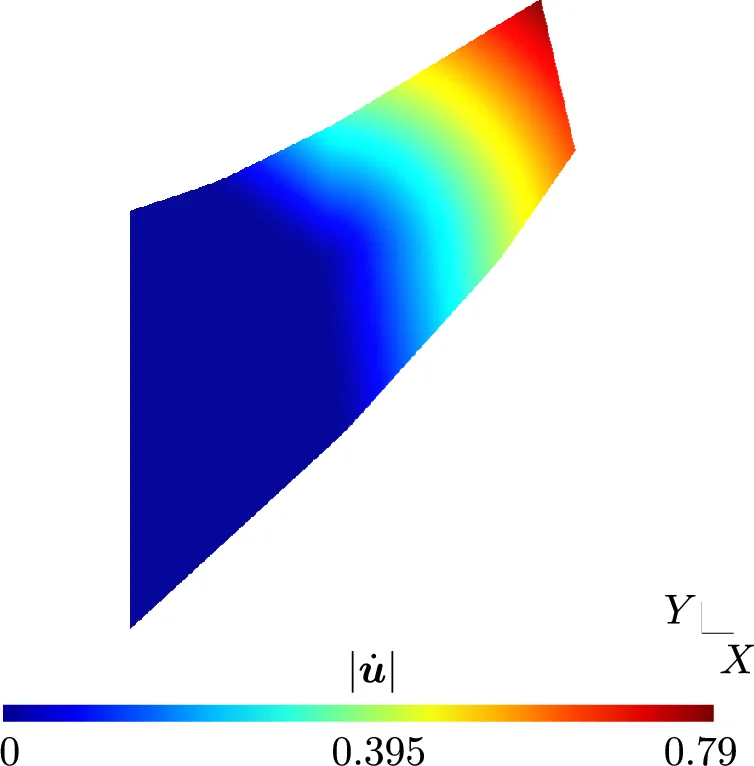
Cook’s membrane—absolute value of velocity at collapse.

The standard Cook’s membrane problem tests an element’s plastic behavior for the case of in-plane shear loading. The problem also tests the effect of mesh distortion. The membrane is fully fixed at *X=0* and loaded by a distributed force at $X=L_1$ (see Fig. 10). The *Z*-component of the displacement is evaluated at the test point A. The plane stress von Mises plasticity model is used, see Eq. (17) for the corresponding dissipation power density. We note that in this in-plane test problem, we eliminate drilling rotations $\varphi _Z$ in the DKMQ24 element because they have no numerical benefit in this example. For other elements the penalty constant is set to $C_{\text {d}}=0.0001$. The numerical results are summarized in Tables 8, and 9 . Let us discuss the results depicted in Fig. 11. We again observe that the most efficient elements are DKMQ24+ and OPQ, which even overcomes results by the Ciria element obtained with the help of adaptive meshing. The Ciria element with adaptive meshing has, on the other hand, as expected, the highest convergence order 2.695 (see Table 23). The highest efficiency is exhibited by the DKMQ24+ quadrangle element. The DKMQ24+ element performs better than the DKMQ24 element in this example. All tested elements converge to the reference solution monotonically from above. The time-dependent results are depicted in Fig. 12. We can observe computational time efficiency of the DKMQ24+ element in this benchmark. The dissipation power and the velocity field at the instance of collapse are depicted in Figs. 13 and 14 respectively.

**Table 8:** Cook’s membrane (triangle elements).

#elements	#dofs	OPT
		*λ* [-]
$2\!\!\times \!\!2\!\!\times \!\!4$	30	0.75546
$4\!\!\times \!\!4\!\!\times \!\!4$	108	0.71277
$8\!\!\times \!\!8\!\!\times \!\!4$	408	0.69485
$16\!\!\times \!\!16\!\!\times \!\!4$	1584	0.68863
$32\!\!\times \!\!32\!\!\times \!\!4$	6240	0.68618
$64\!\!\times \!\!64\!\!\times \!\!4$	24768	0.68524

Reference solution: $\lambda _{\text {ref}}=0.68477$ (DKMQ24+ element at mesh $256\!\times \!256$).

**Table 9:** Cook’s membrane (quad elements).

#elements	#dofs	OPQ	DKMQ24+	#dofs	DKMQ24
		*λ* [-]	*λ* [-]		*λ* [-]
$2\!\!\times \!\!2$	18	0.78278	0.75236	12	1.08481
$4\!\!\times \!\!4$	60	0.72259	0.71510	40	0.84048
$8\!\!\times \!\!8$	216	0.69574	0.69372	144	0.74485
$16\!\!\times \!\!16$	816	0.68857	0.68798	544	0.70739
$32\!\!\times \!\!32$	3168	0.68616	0.68591	2112	0.69315
$64\!\!\times \!\!64$	12480	0.68529	0.68514	8320	0.68783

Reference solution: $\lambda _{\text {ref}}=0.68477$ (DKMQ24+ element at mesh $256\!\times \!256$).

### Simply supported spherical cap under uniform pressure

**Fig. 15:**
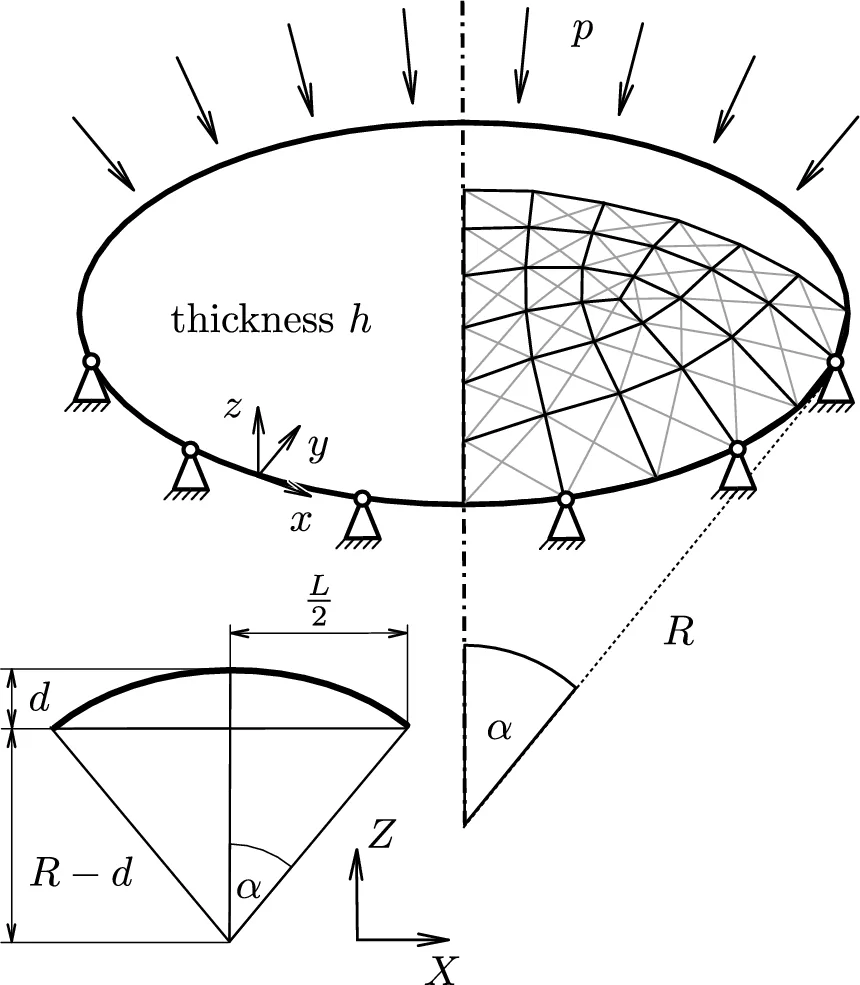
Simply supported spherical cap under uniform pressure with quad mesh $3\times 3\times 3$, a triangular mesh is depicted in gray. Parameters: $R=1.25\,\text {m},\,h=0.001\,\text {m},\,L=2\,\text {m},\,d=R-\sqrt{R^2-\left( \frac{L}{2}\right) ^2}=0.5\,\text {m},\,\alpha =\arcsin \frac{L}{2R}\approx 53.1^{\circ }$, yield strength $\sigma _0=240\,\text {MPa}$, loading pressure $p=1\,\text {Pa}$.

A simply supported spherical cap of thickness *h* loaded by uniform pressure *p* is considered (see Fig. 15). The Tresca failure criterion is considered, see Eq. (20) for the corresponding dissipation power density, and the 3-point Gauss-Lobatto quadrature is used for the integration across the shell thickness. This benchmark is taken from^20^. Only one quarter is computed due to symmetry. Boundary and symmetry conditions are as follows: $u_X=\varphi _Y=\varphi _Z=0$ at *X=0*, $u_Y=\varphi _X=\varphi _Z=0$ at *Y=0*, $u_X=u_Y=u_Z=0$ at *Z=0*. Moreover, $\varphi _y=\varphi _z=0$ is set in the local coordinate system at *Z=0*. The analytical solution $p=\frac{2h\sigma _0}{R}=384.0\,\text {kPa}$ is taken from^52^. The penalty constant is set to $C_{\text {d}}=0.001$. The numerical results are summarized in Tables 10, and 11.

**Table 10:** Simply supported spherical cap under uniform pressure (triangle elements).

#elements	#dofs	Specht (OPT)	DKT (OPT)	DKMT (OPT)
		*λ* [-]	*λ* [-]	*λ* [-]
$3\!\!\times \!\!3\!\!\times \!\!3\!\!\times \!\!4$	324	166.67	247.48	245.42
$6\!\!\times \!\!6\!\!\times \!\!3\!\!\times \!\!4$	1296	179.37	285.71	283.03
$12\!\!\times \!\!12\!\!\times \!\!3\!\!\times \!\!4$	5184	263.55	329.82	328.53
$24\!\!\times \!\!24\!\!\times \!\!3\!\!\times \!\!4$	20736	355.36	369.66	373.46
$48\!\!\times \!\!48\!\!\times \!\!3\!\!\times \!\!4$	82944	373.19	381.25	381.04

Reference solution: $$\lambda _{\text {ref}}=384.00$$ (^52^).

**Table 11:** Simply supported spherical cap under uniform pressure (quad elements).

#elements	#dofs	DKQ (OPQ)	DKMQ (OPQ)	DKMQ24	DKMQ24+
		*λ* [-]	*λ* [-]	*λ* [-]	*λ* [-]
$3\!\!\times \!\!3\!\!\times \!\!3$	162	334.68	335.44	375.68	340.14
$6\!\!\times \!\!6\!\!\times \!\!3$	648	353.76	354.14	379.78	354.82
$12\!\!\times \!\!12\!\!\times \!\!3$	2592	371.71	367.65	382.41	369.29
$24\!\!\times \!\!24\!\!\times \!\!3$	10368	380.62	378.25	383.43	380.73
$48\!\!\times \!\!48\!\!\times \!\!3$	41472	382.98	381.73	383.83	382.96

Reference solution: $$\lambda _{\text {ref}}=384.00$$ (^52^).

**Fig. 16:**
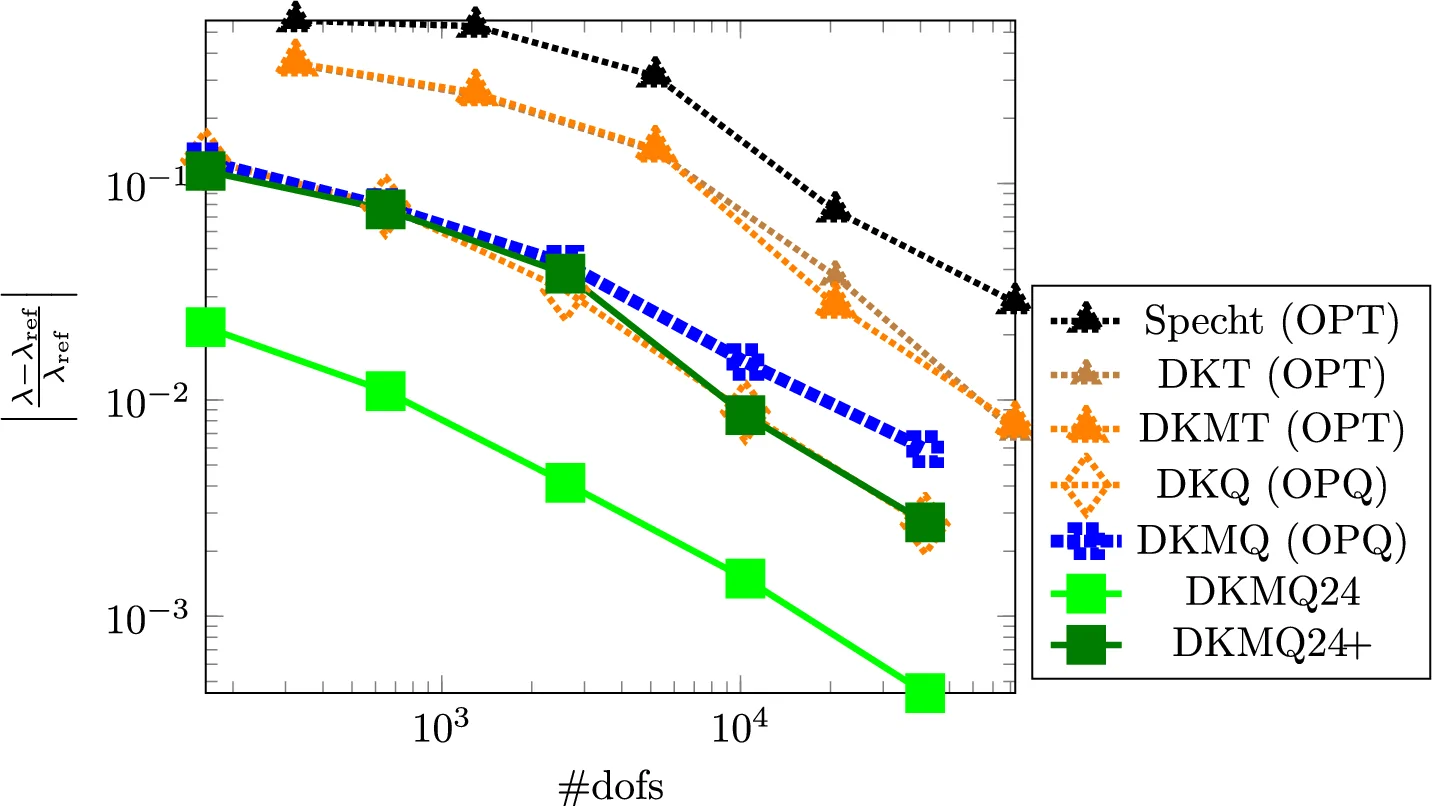
Simply supported spherical cap under uniform pressure.

**Fig. 17:**
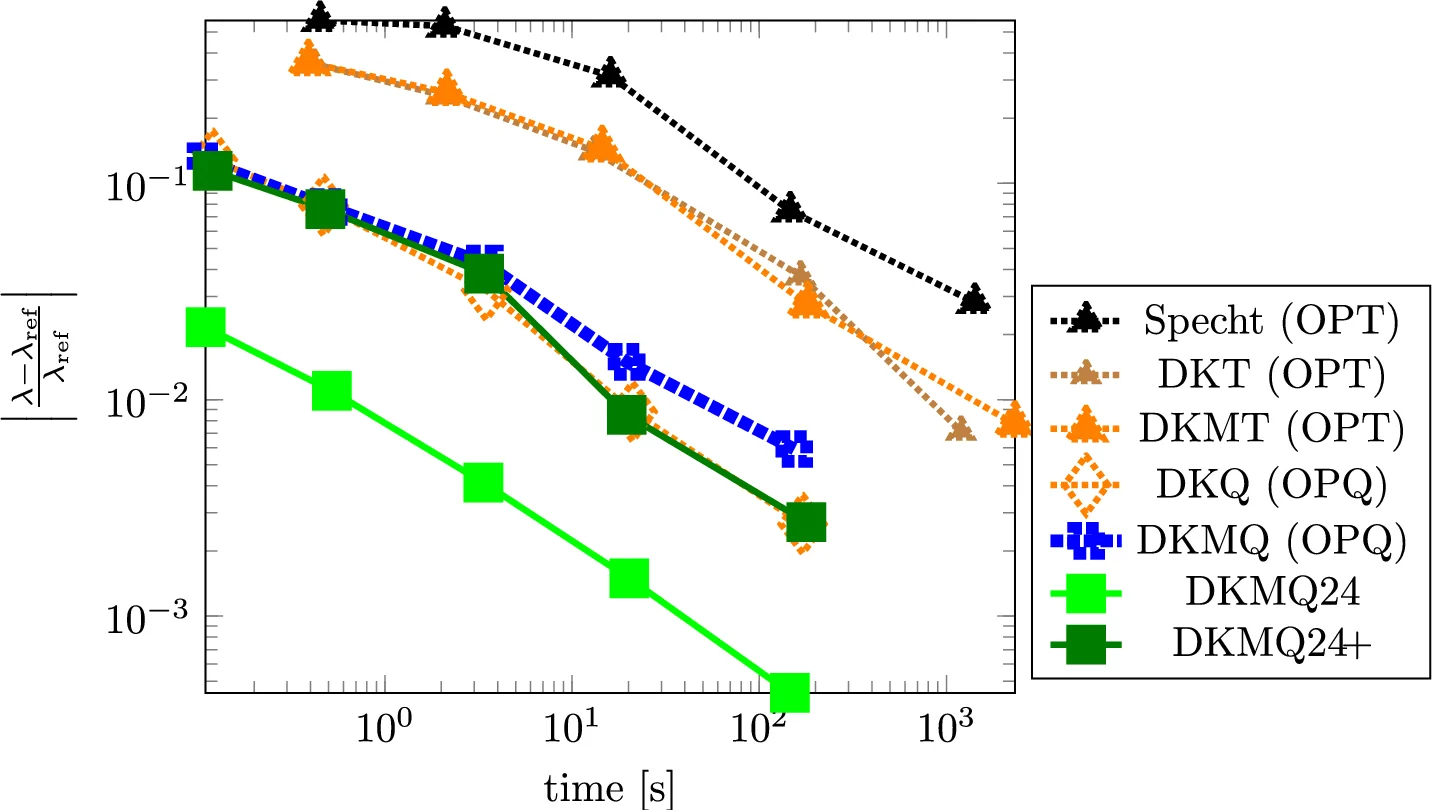
Simply supported spherical cap under uniform pressure.

**Fig. 18:**
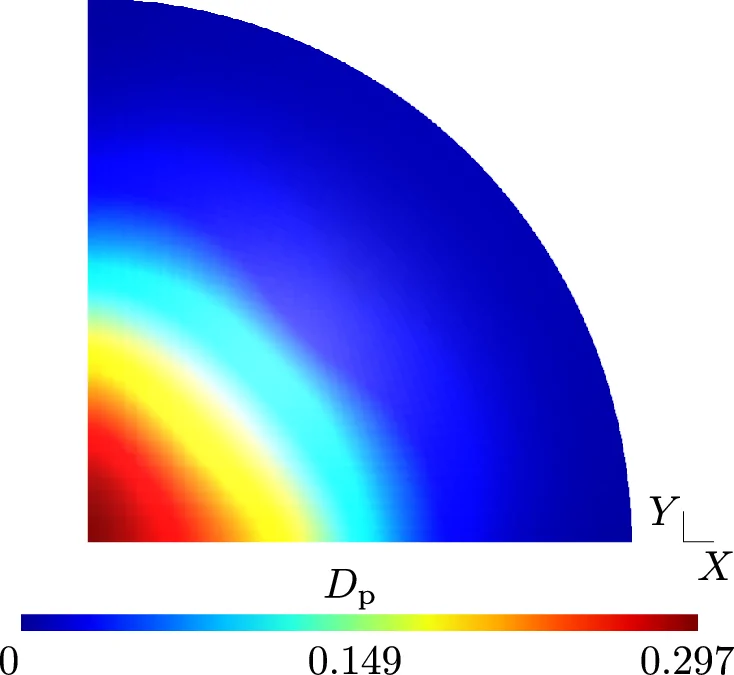
Simply supported spherical cap under uniform pressure—dissipation power at collapse (DKMQ24 element).

**Fig. 19:**
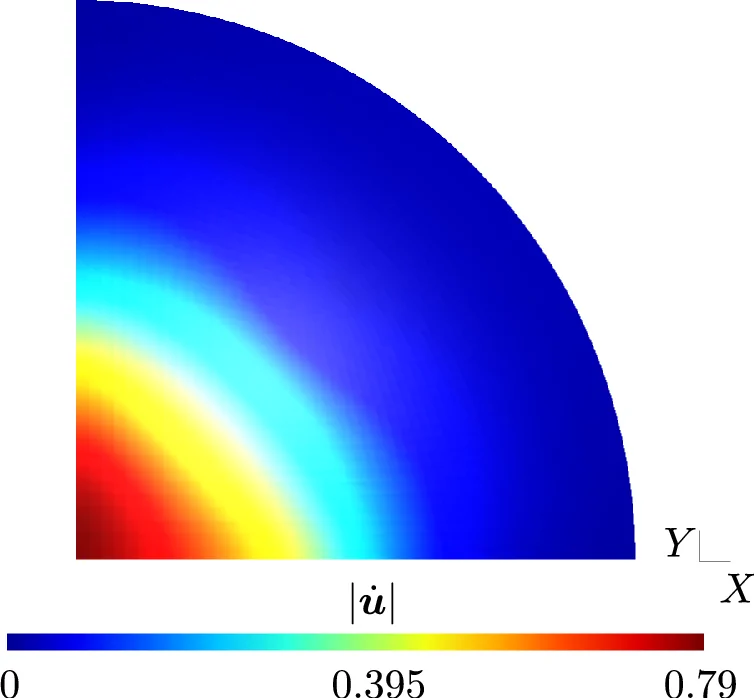
Simply supported spherical cap under uniform pressure—absolute value of velocity at collapse (DKMQ24 element).

Let us discuss the results depicted in Fig. 16. Quad elements are clearly superior to all triangle elements. The most efficient element is DKMQ24, which outperforms the DKMQ24+ element in this example. Contrary to other benchmark problems in this paper, all the tested elements converge to the reference solution from below. The time-dependent results are depicted in Fig. 17. The dissipation power and the velocity field at the instance of collapse are depicted in Figs. 18 and 19 respectively.

### Clamped cylindrical tube under vertical distributed loading

**Fig. 20:**
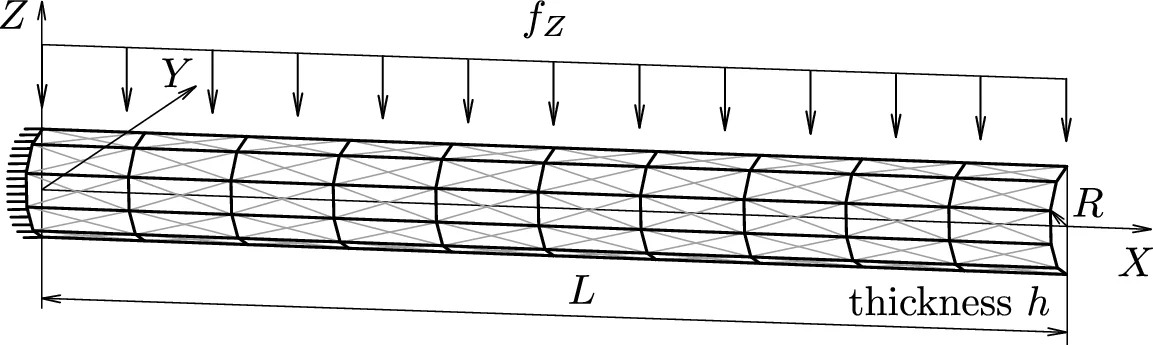
Clamped cylindrical tube under vertical distributed loading with a uniform quad mesh of *10× 5*, a triangular mesh is depicted in gray. Parameters: $R=0.05\,\text {m},\,h=0.001\,\text {m},\,L=2\,\text {m}$, yield strength $\sigma _0=240\,\text {MPa}$, the total loading force is $1\,\text {N}$.

**Fig. 21:**
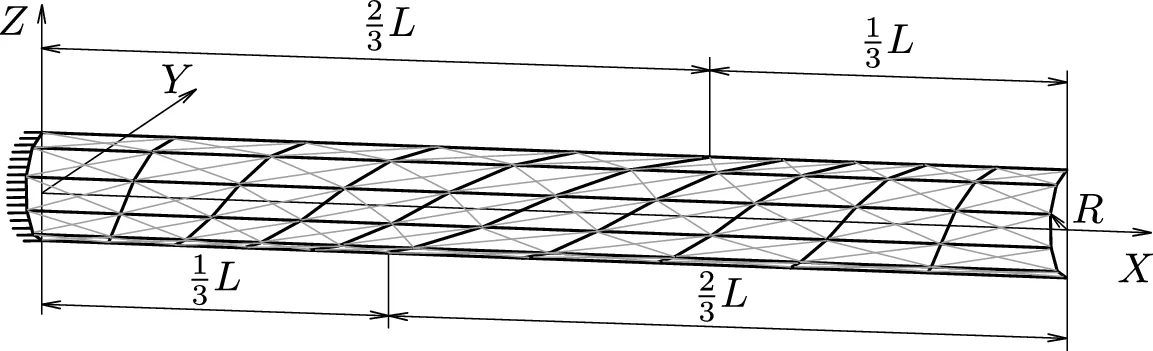
Clamped cylindrical tube with a distorted quad mesh of *10× 5*. A triangular mesh is depicted in gray.

A clamped cylindrical tube of length 2*L*, thickness *h* and radius *R* under vertical distributed loading $f_Z$ is considered (see Fig. 20). The behavior on both uniform mesh (see Fig. 20) and the distorted mesh (see Fig. 21) is investigated. The Ilyushin failure criterion with yield stress $\sigma _0$ is considered, see Eqs. (13-16) for the corresponding dissipation power density. This benchmark is taken from^20^. Only one quarter is computed due to symmetry. Boundary and symmetry conditions are as follows: $u_X=u_Y=u_Z=\varphi _X=\varphi _Y=\varphi _Z=0$ at *X=0*, $u_X=\varphi _Y=\varphi _Z=0$ at *X=L=2*, $u_Y=\varphi _X=\varphi _Z=0$ at *Y=0*. The problem has the following approximate analytical solution based on the beam theory (fully valid for $h\ll R\ll 2L$): the ultimate line load on the rigid clamped beam of length 2*L* is $q=16\frac{M}{{(2L)}^{2}}\,\left[ \frac{\text {N}}{\text {m}}\right]$, where the ultimate moment is given by $M \approx 4hR^2\sigma _0\,\left[ \text {Nm}\right]$^20^. The ultimate load on the simulated quarter of the tube is given by $F_{Z,\text {ult}}=\frac{qL}{2}$, which gives us the final formula

51 $$\begin{aligned} F_{Z,\text {ult}}=8\frac{hR^2\sigma _0}{L} = 4800\,\text {N}. \end{aligned}$$

**Fig. 22:**
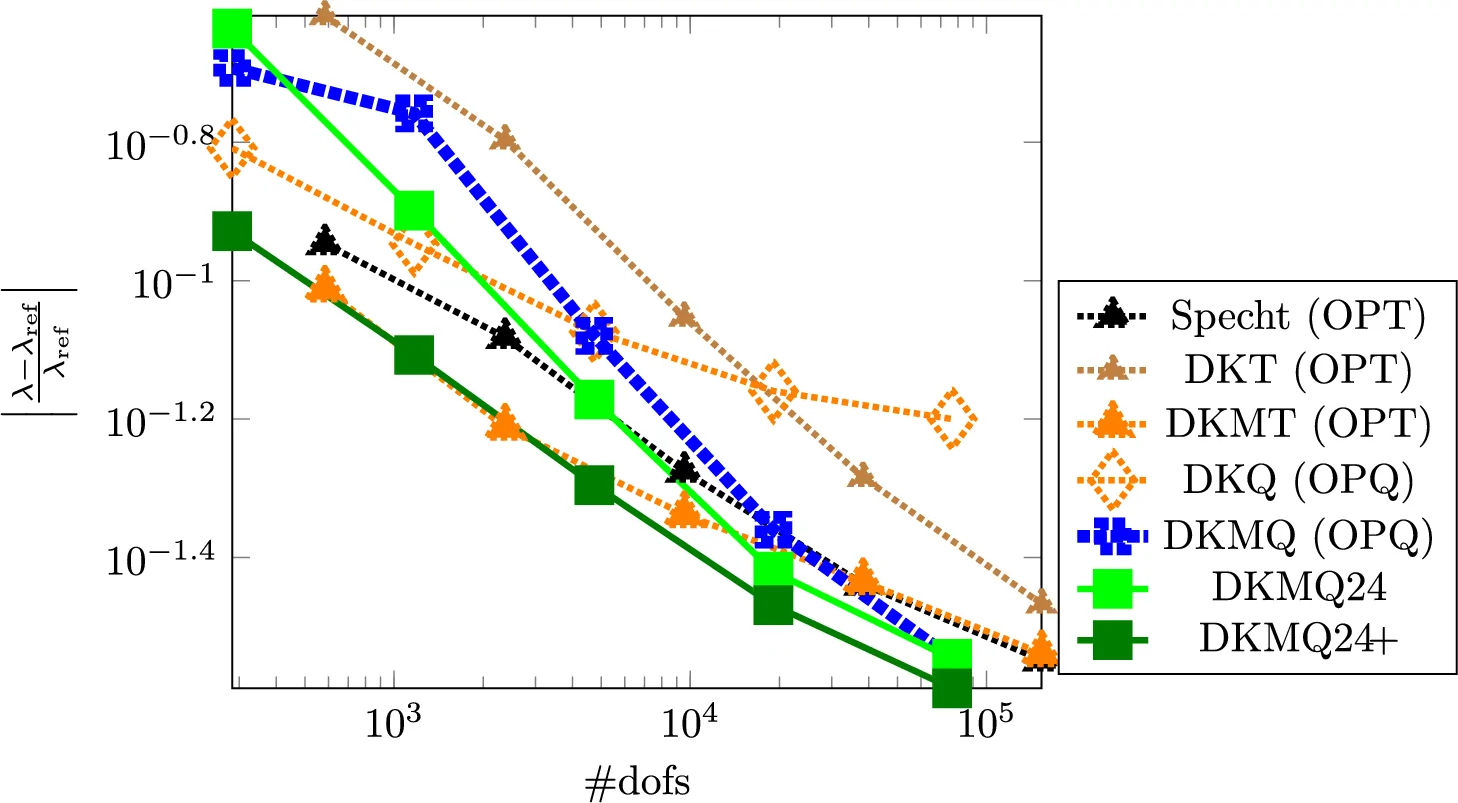
Clamped cylindrical tube, uniform mesh.

**Fig. 23:**
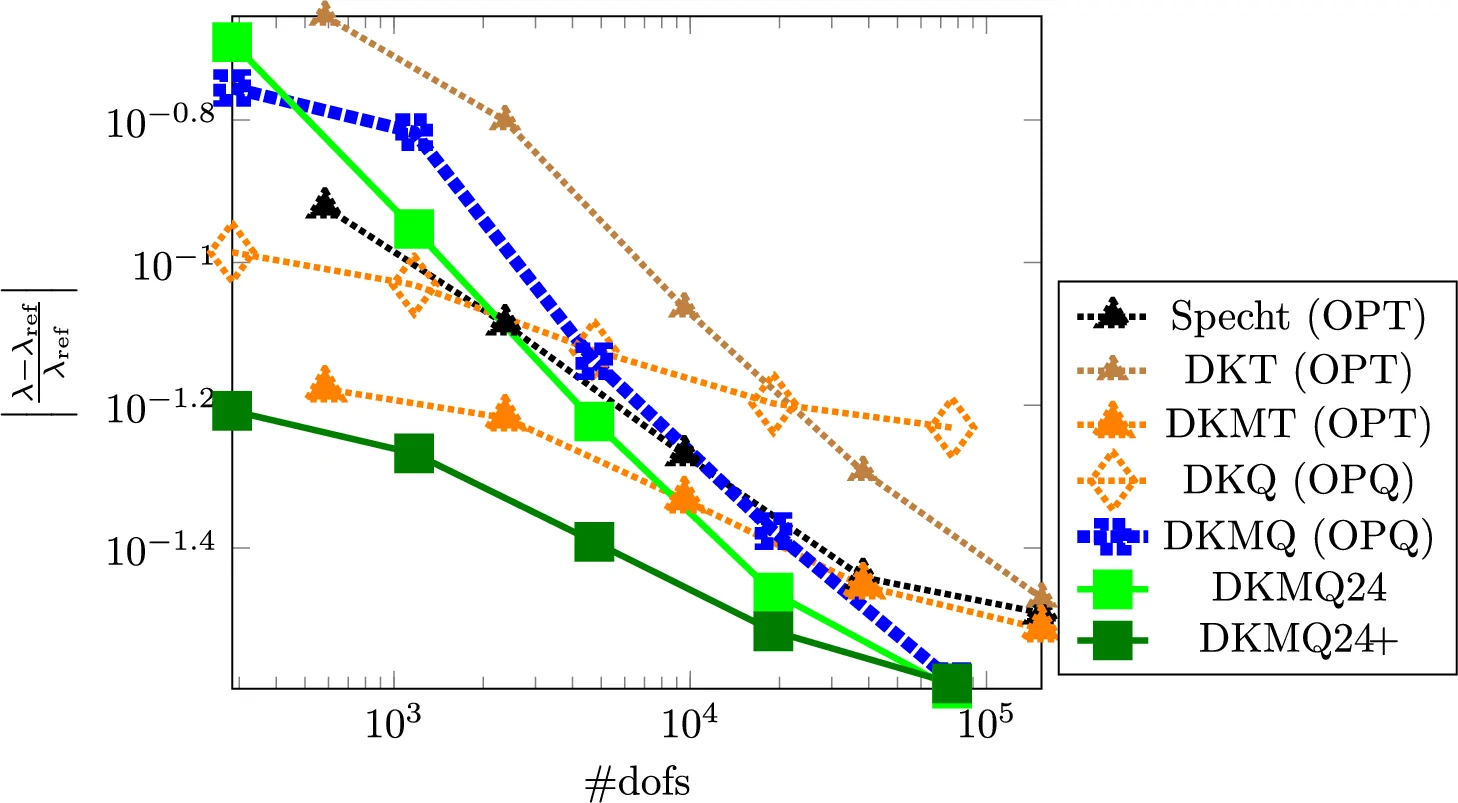
Clamped cylindrical tube, distorted mesh.

**Fig. 24:**
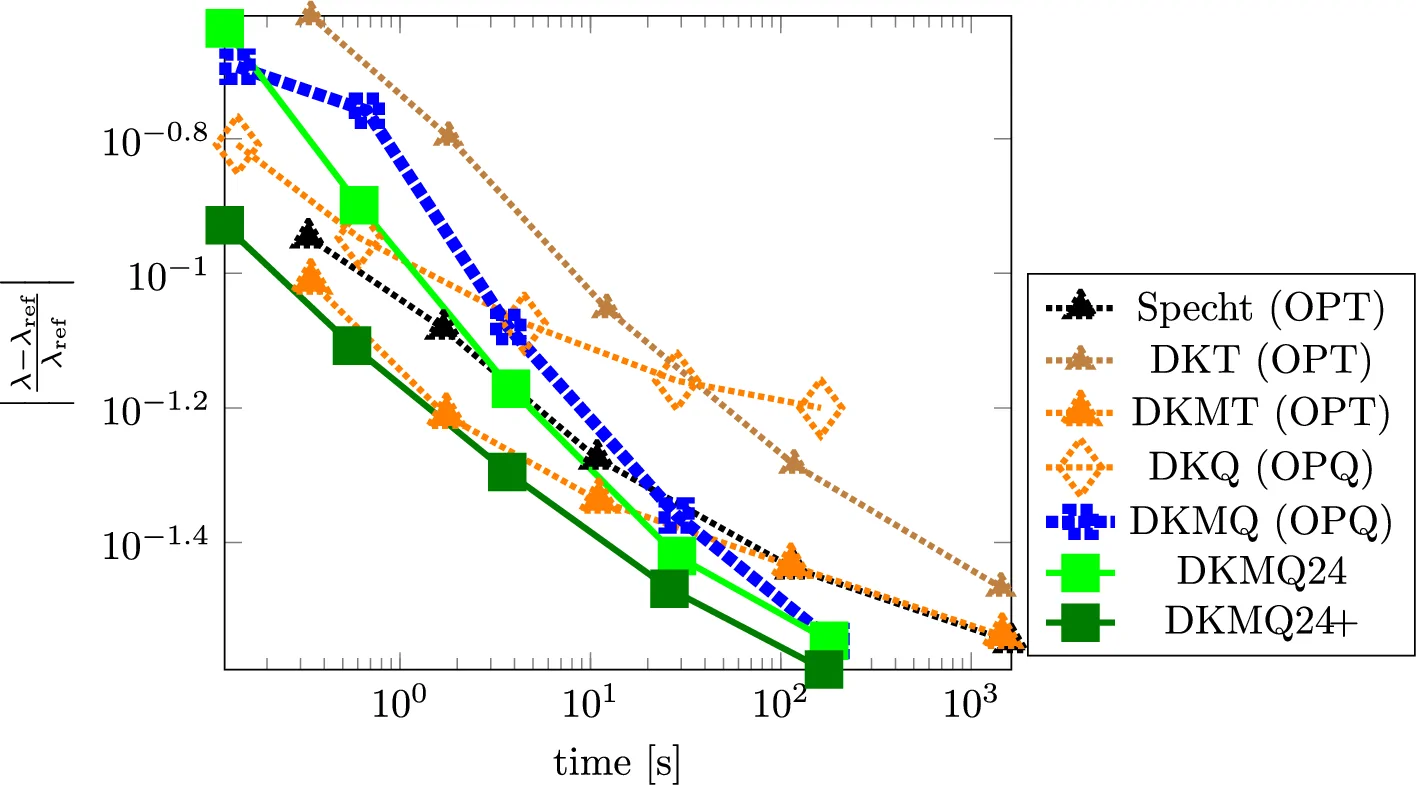
Clamped cylindrical tube, time-dependent results, uniform mesh.

**Fig. 25:**
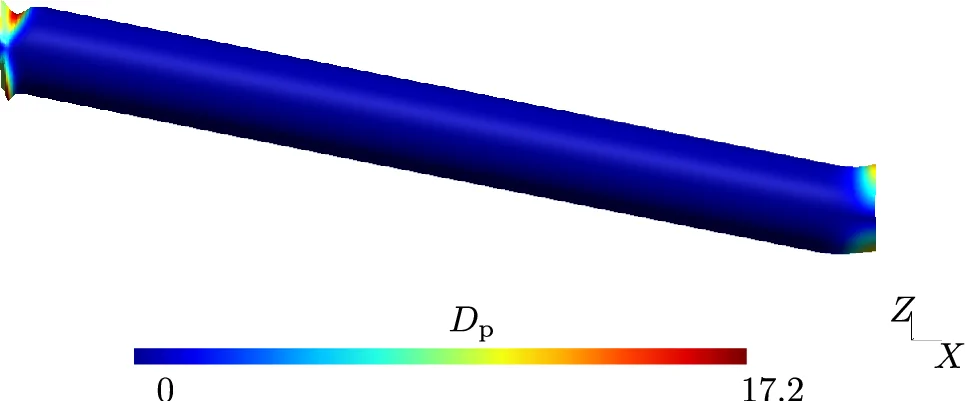
Clamped cylindrical tube—dissipation power at collapse (DKMQ24 element).

**Fig. 26:**
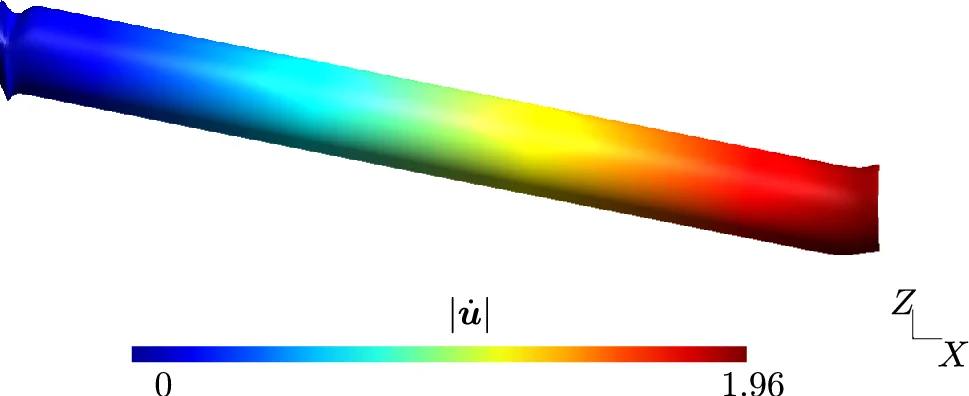
Clamped cylindrical tube—absolute value of velocity at collapse (DKMQ24 element).

The penalty constant is set to $C_{\text {d}}=0.001$ in this example. The numerical results for the case of the uniform mesh are summarized in Tables 12, and 13 and in Fig. 22. The numerical results for the case of the distorted mesh are summarized in Tables 14 and 15 and in Fig. 23. Let us first discuss the results depicted in Fig. 22. The DKQ (OPQ) element does not perform well; the situation worsens for smaller tube radii (we have tried $R=0.03\,\text {m}$). The most efficient elements in this example are the quadrangles DKMQ24, and DKMQ24+ (both with respect to the number of degrees of freedom and computational time). DKMQ24+ outperforms the former two elements. All tested elements converge monotonically from above to the reference solution. The numerical results summarized in Tables 14 and 15 and shown in Fig. 23 also demonstrate the convergence of all tested elements to the reference solution. The time-dependent results are depicted in Fig. 24, the most efficient element is DKMQ24+. The dissipation power and the velocity field at the instance of collapse are depicted in Figs. 25 and 26 respectively.

**Table 12:** Clamped cylindrical tube (triangle elements), uniform mesh.

#elements	#dofs	Specht (OPT)	DKT (OPT)	DKMT (OPT)
		*λ* [-]	*λ* [-]	*λ* [-]
$10\!\!\times \!\!5\!\!\times \!\!4$	284	5343.0	5958.2	5266.9
$20\!\!\times \!\!10\!\!\times \!\!4$	1169	5197.5	5566.3	5094.9
$40\!\!\times \!\!20\!\!\times \!\!4$	4739	5054.7	5224.9	5020.5
$80\!\!\times \!\!40\!\!\times \!\!4$	19079	4974.9	5049.5	4976.5
$160\!\!\times \!\!80\!\!\times \!\!4$	76559	4935.9	4963.8	4938.9

Reference solution: $\lambda _\text {ref}=4800.0$ (approximate analytical solution).

**Table 13:** Clamped cylindrical tube (quad elements), uniform mesh.

#elements	#dofs	DKQ (OPQ)	DKMQ (OPQ)	DKMQ24	DKMQ24+
		*λ* [-]	*λ* [-]	*λ* [-]	*λ* [-]
$10\!\!\times \!\!5$	284	5545.8	5773.6	5910.1	5365.9
$20\!\!\times \!\!10$	1169	5342.3	5637.3	5405.9	5174.9
$40\!\!\times \!\!20$	4739	5204.9	5199.5	5123.3	5043.2
$80\!\!\times \!\!40$	19079	5132.5	5009.6	4982.4	4963.1
$160\!\!\times \!\!80$	76559	5102.9	4936.4	4936.5	4923.7

Reference solution: $\lambda _\text {ref}=4800.0$ (approximate analytical solution).

**Table 14:** Clamped cylindrical tube (triangle elements), distorted mesh.

#elements	#dofs	Specht (OPT)	DKT (OPT)	DKMT (OPT)
		*λ* [-]	*λ* [-]	*λ* [-]
$10\!\!\times \!\!5\!\!\times \!\!4$	284	5374.4	5863.5	5247.1
$20\!\!\times \!\!10\!\!\times \!\!4$	1169	5193.8	5558.8	5090.7
$40\!\!\times \!\!20\!\!\times \!\!4$	4739	5058.1	5214.5	5023.2
$80\!\!\times \!\!40\!\!\times \!\!4$	19079	4973.7	5044.5	4969.0
$160\!\!\times \!\!80\!\!\times \!\!4$	76559	4954.9	4962.6	4947.2

Reference solution: $\lambda _\text {ref}=4800.0$ (approximate analytical solution).

**Table 15:** Clamped cylindrical tube (quad elements), distorted mesh.

#elements	#dofs	DKQ (OPQ)	DKMQ (OPQ)	DKMQ24	DKMQ24+
		*λ* [-]	*λ* [-]	*λ* [-]	*λ* [-]
$10\!\!\times \!\!5$	284	5296.3	5643.5	5777.9	5097.5
$20\!\!\times \!\!10$	1169	5246.7	5531.4	5335.0	5059.3
$40\!\!\times \!\!20$	4739	5161.4	5149.1	5087.4	4994.8
$80\!\!\times \!\!40$	19079	5104.6	5001.7	4965.4	4946.0
$160\!\!\times \!\!80$	76559	5081.7	4924.3	4921.3	4923.4

Reference solution: $\lambda _\text {ref}=4800.0$ (approximate analytical solution).

The behavior of the tested elements on the distorted mesh shown in Fig. 21 is also explored. The numerical results, summarized in Tables 14 and 15 and shown in Fig. 23, demonstrate the convergence of all tested elements to the reference solution.

### Block with asymmetric holes

**Fig. 27:**
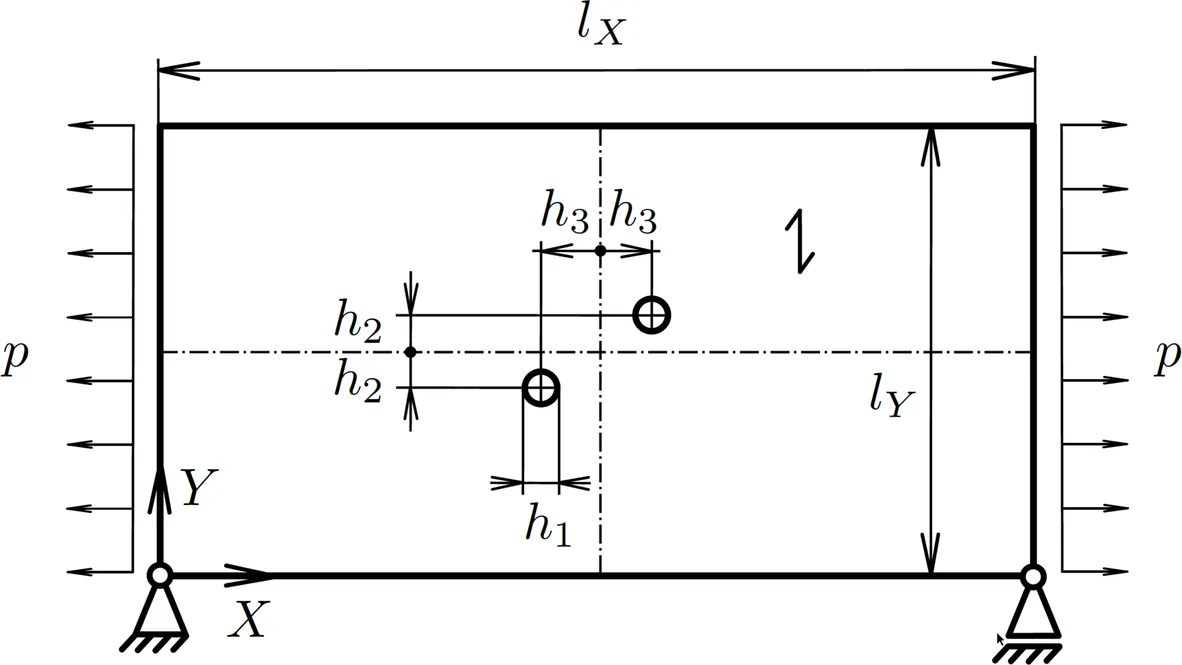
Block with asymmetric holes. Parameters: $l_X=0.1\,\text {m},\, l_Y=0.05\,\text {m},\, h_1=4\,\text {mm},\, h_2=2.5\,\text {mm},\, h_3=5.5\,\text {mm}, p=1\,\text {MPa}$, strengths (recalculated from^16^) $f_{\text {t}x}=3.3\,\text {MPa}$, $f_{\text {c}x}=4.6\,\text {MPa}$, $f_{\text {t}y}=79\,\text {MPa}$, $f_{\text {c}y}=52\,\text {MPa}$, $f_{\text {v}xy}=9.2\,\text {MPa}$.

**Fig. 28:**
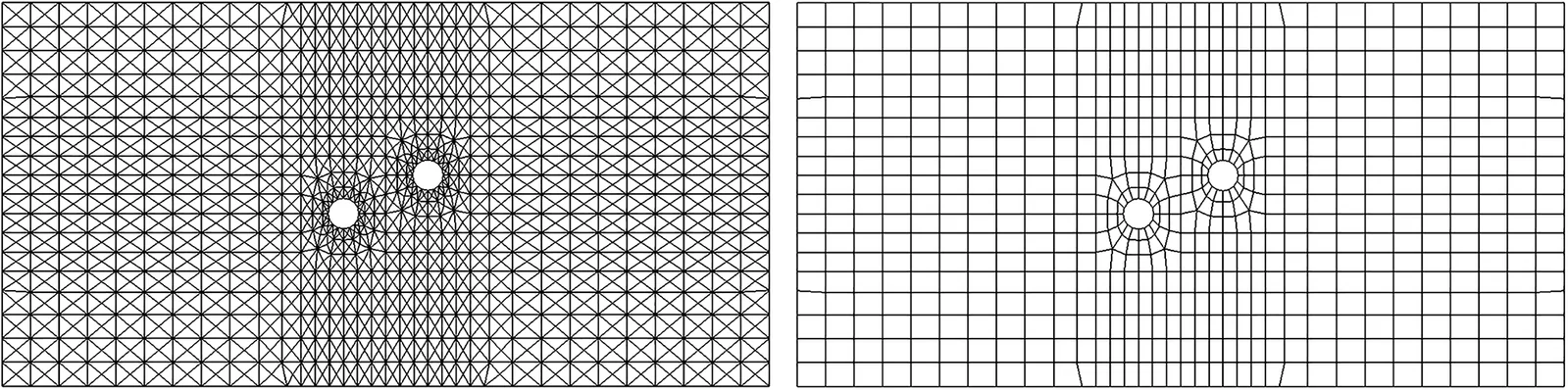
Block with asymmetric holes—left: triangular mesh with 2576 elements, right: quad mesh with 644 elements.

**Fig. 29:**
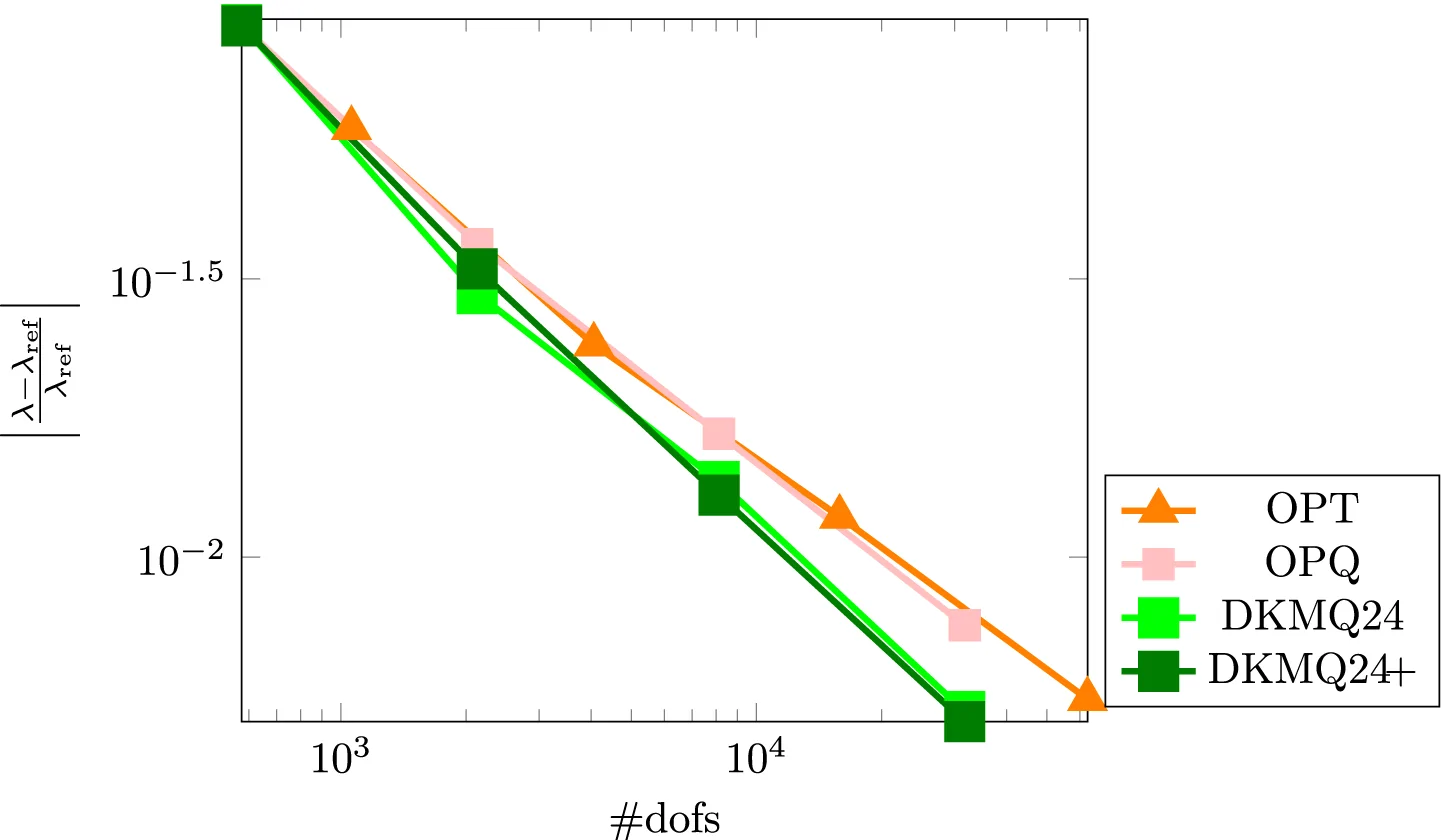
Block with asymmetric holes.

**Fig. 30:**
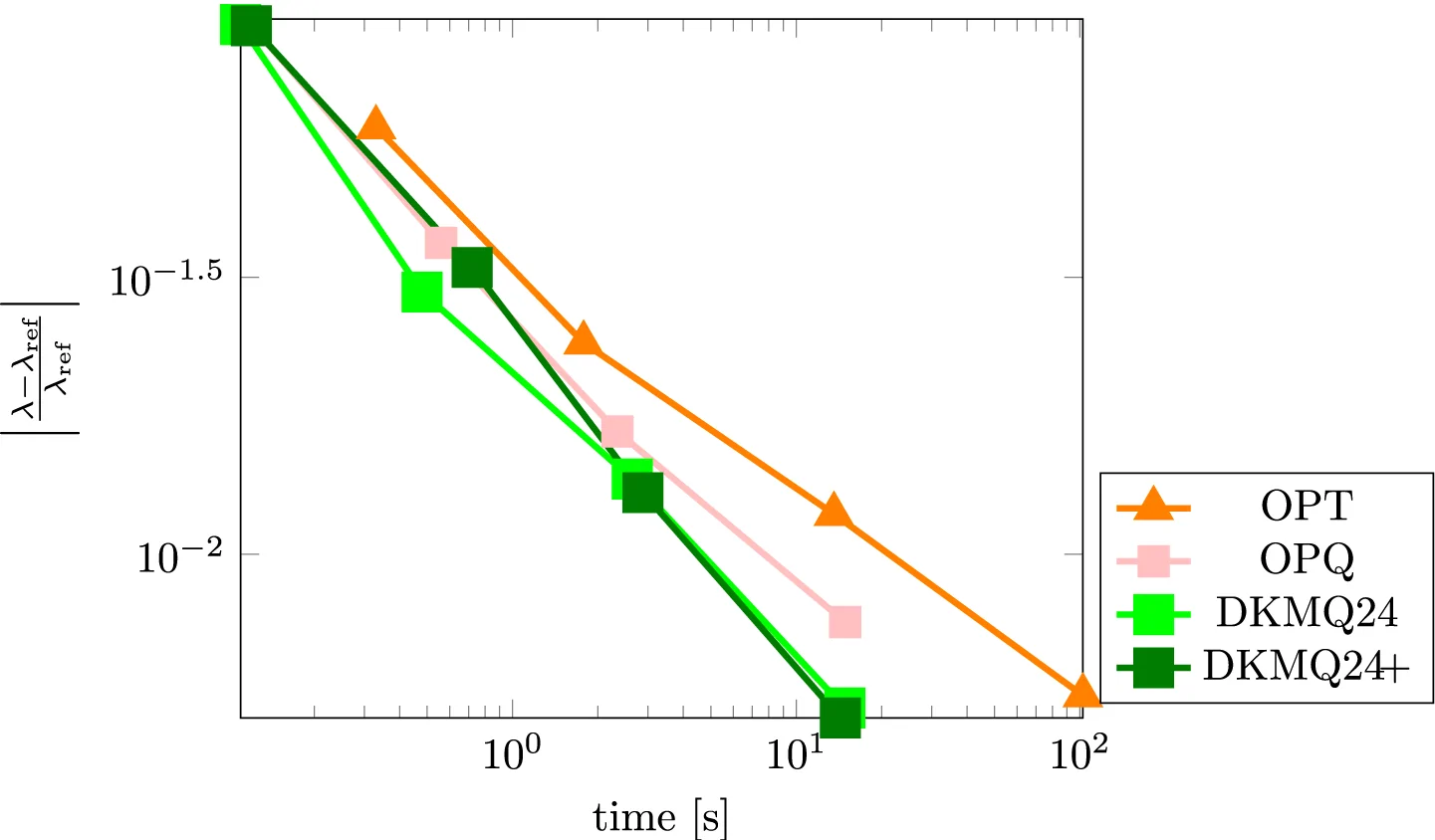
Block with asymmetric holes.

**Fig. 31:**
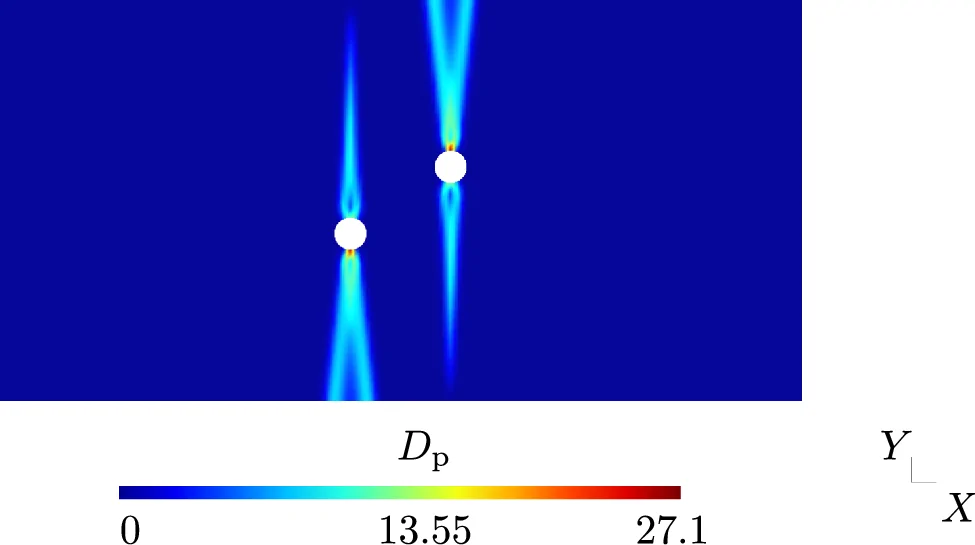
Block with asymmetric holes—dissipation power at collapse.

**Fig. 32:**
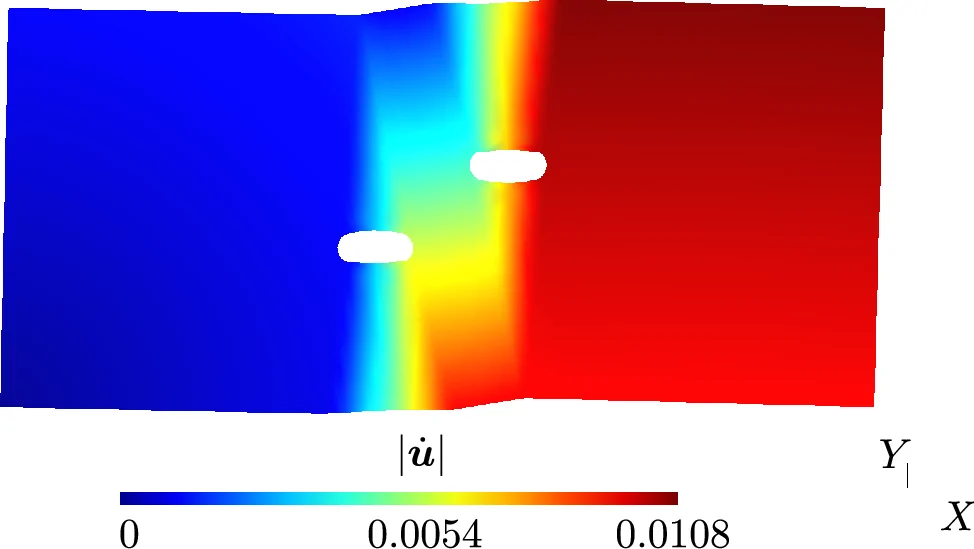
Block with asymmetric holes—velocity field at collapse.

The block with asymmetric holes under tensile loading, a commonly-used benchmark for FELA, was first studied by Zouain et al. in 2002^41^. The block of length $l_X$, height $h_Y$ and thickness *h* is subjected to pressure *p* in *X*, see Fig. 27. Boundary and symmetry conditions are as follows: $u_X=u_Y=u_Z=\varphi _X=\varphi _Y=\varphi _Z=0$ at *[X,Y]=[0,0]*, $u_Y=u_Z=\varphi _X=\varphi _Y=\varphi _Z=0$ at $[X,Y]=[l_X,0]$ and $u_Z=\varphi _X=\varphi _Y=0$ everywhere. The plane strain orthotropic Tsai-Wu failure criterion with fibers oriented in the *Y* direction is considered, with strengths values taken from^15^. For the corresponding dissipation power density see Eq. (22). Since the analytical solution is not available, we use Aitken’s extrapolation of the three finest results of the DKMQ24+ element as the reference *(λ =3.1072)* which seems to be most probable limit value in this example. It should be noted that the solution given in^16^ for this plane strain problem is calculated approximately on solid elements with double-symmetric conditions in the *Y*-direction and therefore converges to a slightly smaller value (*λ =3.082*). We use higher values of the drilling rotation stabilization in this example $C_{\text {d}}=0.1$. The mesh used is shown in Fig. 28, and the results are summarized in Tables 16 and 17. Let us discuss the results depicted in Fig. 29. Quadrangle elements again outperform all tested triangle elements, in particular the elements DKMQ24, and DKMQ24+. All tested elements converge monotonically from above to the reference solution. The time-dependent results are depicted in Fig. 30. We can observe computational time efficiency of the DKMQ24, and DKMQ24+ elements in this benchmark. The dissipation power and the velocity field at the instance of collapse are depicted in Figs. 31 and 32 respectively.

**Table 16:** Block with asymmetric holes (triangle elements).

#elements	#dofs	OPT
		*λ* [-]
644	1060	3.2905
2576	4060	3.1821
10304	15856	3.1439
41216	62632	3.1245

Reference solution: $\lambda _\text {ref}=3.1072$ (Aitken’s extrapolation of the DKMQ24+ results).

**Table 17:** Block with asymmetric holes (quad elements).

#elements	#dofs	OPQ	DKMQ24	DKMQ24+
		*λ* [-]	*λ* [-]	*λ* [-]
161	577	3.3876	3.3884	3.3870
644	2128	3.2206	3.1996	3.2096
2576	8128	3.1589	3.1497	3.1473
10304	31720	3.1306	3.1236	3.1229

Reference solution: $\lambda _\text {ref}=3.1072$ (Aitken’s extrapolation of the DKMQ24+ results).

### Simply supported plate in the sequential limit analysis

**Fig. 33:**
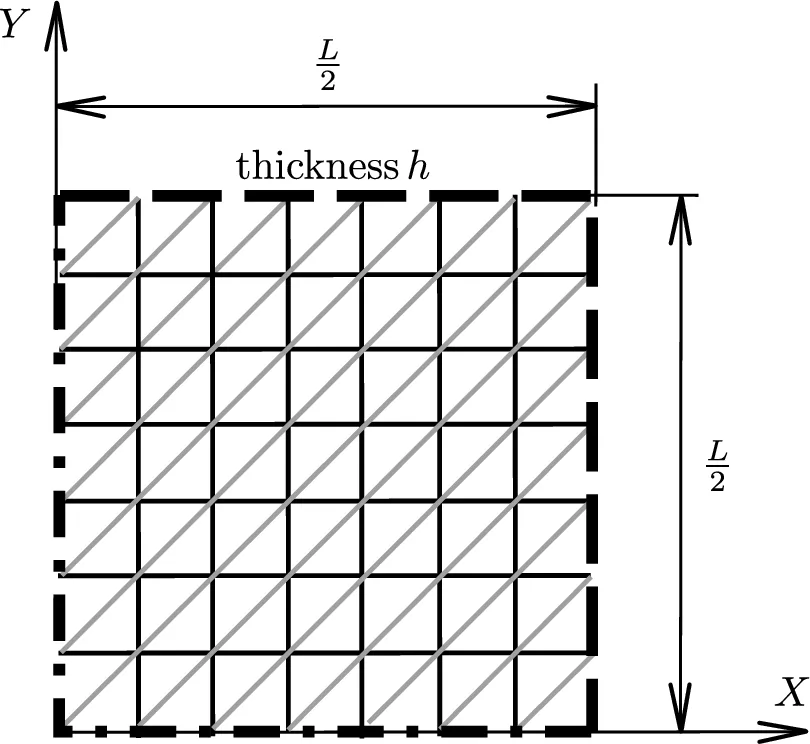
Simply supported pressure loaded square plate with quad mesh $7\!\times \!7$. The triangular mesh is depicted in gray. Parameters: $L=20\,\text {mm},\,h=0.2\,\text {mm}$, yield strength $\sigma _0=200\,\text {MPa}$, loading pressure $p=20\,\text {kPa}$.

A simply supported, pressure-loaded square plate is considered in the SFELA (see Fig. 33). Only one quarter is computed due to its symmetry. Symmetry conditions are as follows: $u_X=\varphi _Y=\varphi _Z=0$ at *X=0* and $u_Y=\varphi _X=\varphi _Z=0$ at *Y=0*. Two different boundary conditions are used:

1. $u_X=u_Y=u_Z=\varphi _X=\varphi _Z=0$ at $X=\frac{L}{2}$ and $u_X=u_Y=u_Z=\varphi _Y=\varphi _Z=0$ at $Y=\frac{L}{2}$ (hard simply supported conditions)
2. $u_Z=\varphi _X=\varphi _Z=0$ at $X=\frac{L}{2}$ and $u_Z=\varphi _Y=\varphi _Z=0$ at $Y=\frac{L}{2}$ (hard simply supported conditions together with free $u_X$ and $u_Y$, which represents in-plane sliding of supports)

**Fig. 34:**
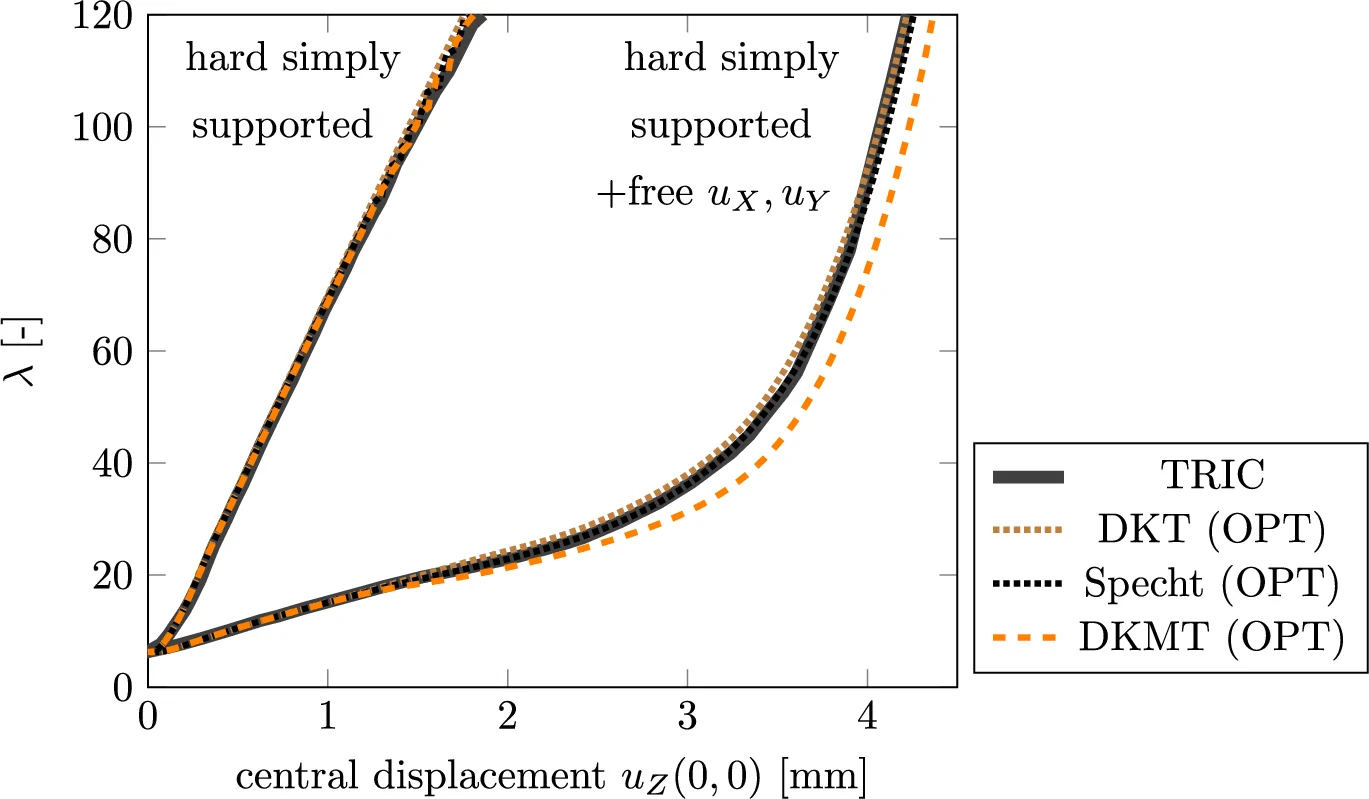
Sequential limit analysis of a simply supported plate, two types of boundary conditions are tested: 1. hard simply supported, and 2. hard simply supported with free $u_X$ and $u_Y$. The step is 0.0325 mm. Comparison of triangle elements.

**Fig. 35:**
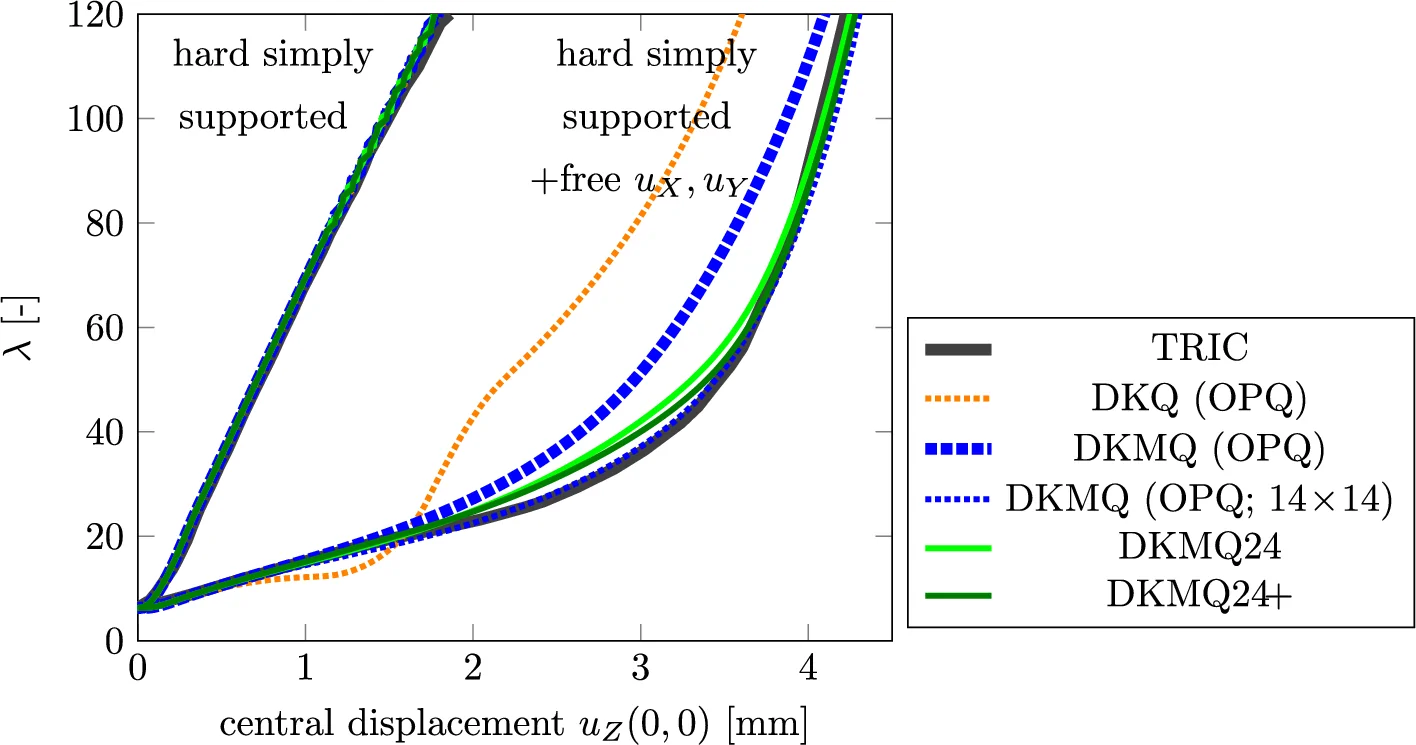
Sequential limit analysis of a simply supported plate, two types of boundary conditions are tested: 1. hard simply supported, and 2. hard simply supported with free $u_X$ and $u_Y$. The step is 0.0325 mm. Comparison of the TRIC and quadrangle elements.

**Fig. 36:**
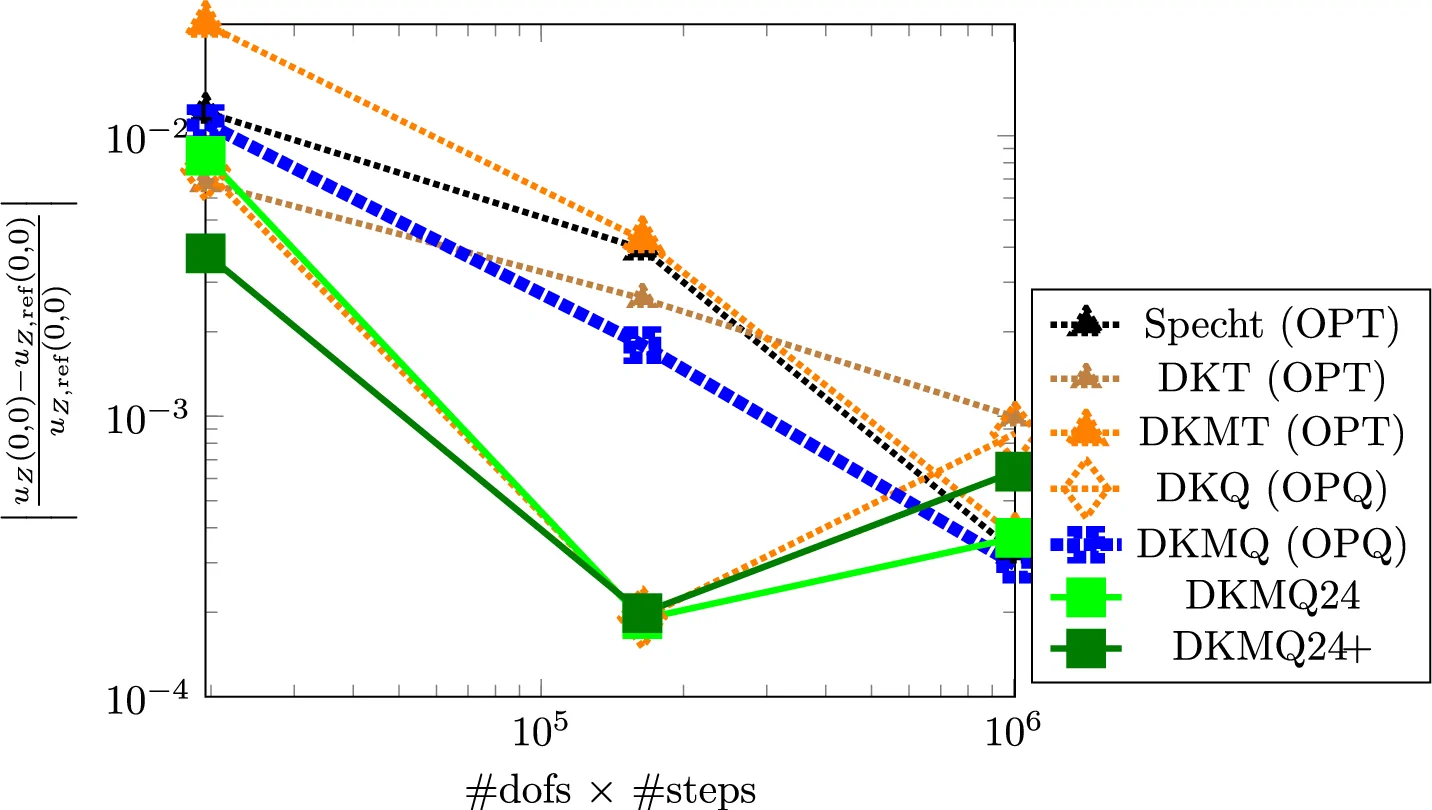
Sequential limit analysis of a simply supported plate under Case 1 boundary conditions. Error in the central displacement $u_Z(0,0)$ at *λ =120*.

**Fig. 37:**
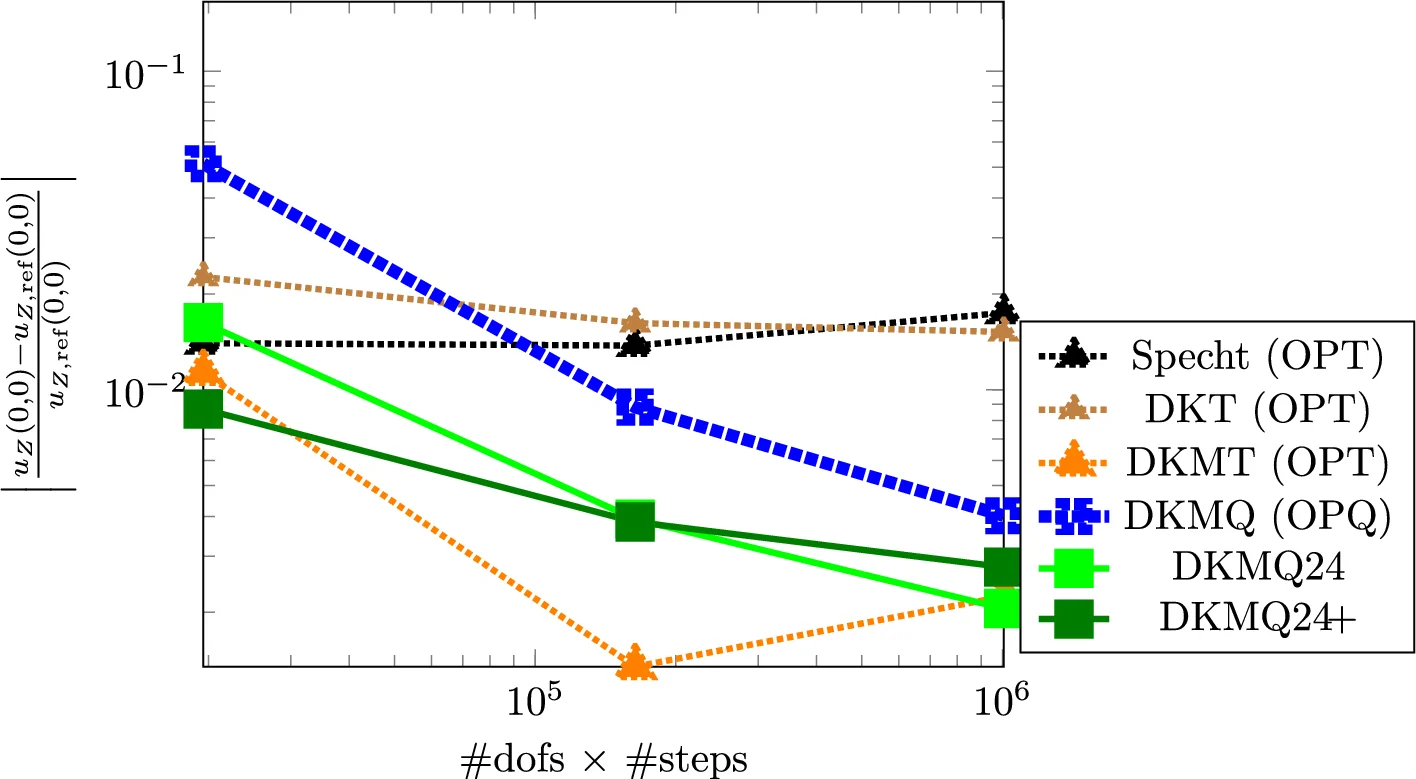
Sequential limit analysis of a simply supported plate under Case 2 boundary conditions. Error in the central displacement $u_Z(0,0)$ at *λ =120*. The DKQ (OPQ) element is not shown due to its divergence.

The Ilyushin stress-resultant plasticity model is considered; see Eqs. (13)-(16) for the corresponding dissipation power density. The penalty constant is set to $C_{\text {d}}=0.001$ in this example. Since an analytical solution for this case is not available, our simulations are compared with the results of Corradi (see Fig. 5b in^30^), obtained using the TRIC shell element^43^. In this analysis, we employed a $7\!\times \!7\!\times \!2$ mesh for triangular elements and a $7\!\times \!7$ mesh for quadrilateral elements (see Fig. 33). The results for both boundary conditions are shown in Figs. 34 and 35. We also perform a more detailed quantitative analysis for both types of boundary conditions, in which the vertical displacement at point *[0,0]* for *λ=120* is compared across various mesh and step sizes, with finer meshes consistently paired with finer step sizes. The numerical results of this analysis for Case 1 boundary conditions (hard simply supported) are presented in Tables 18, 19, and Fig. 36, whereas the results for Case 2 boundary conditions (hard simply supported with free $u_X,~u_Y$) are presented in Tables 20, 21, and Fig. 37. Let us first comment on the results given in Figs. 34 and 35. Under boundary condition 1, all elements yield practically identical results compared with the TRIC element. Under boundary condition 2, the DKQ (OPQ) element exhibits a large error, following a trajectory different from that of the TRIC element. This error is not reduced on finer meshes, even when the $84\!\times \!84$ mesh was used. Under the same boundary condition, the DKMQ (OPQ) element requires a $14\!\times \!14$ mesh to achieve accuracy comparable to other shell elements (as is also clearly shown in Table 21). This reduced accuracy is attributed to the limitations of the rigid-links correction (warping matrix). All other elements yield practically identical results compared with Corradi’s TRIC element (except for the DKMT (OPT) element, which delivers slightly higher results; however, it yields the same value as the DKMQ (OPQ), DKMQ24, and DKMQ24+ elements, as can be seen in Tables 20 and 21). Let us now comment on the results presented in Tables 18, 19, 20, and 21 and in Figs. 36 and 37. Under boundary condition 1, all elements demonstrate convergence as both mesh size and step size approach zero. Moreover, quadrilateral elements generally exhibit lower error compared with triangular elements. Under boundary condition 2, we again observe divergence of the DKQ (OPQ) element. Under the same boundary condition, the DKMT element and all quadrilateral elements converge to the same value, whereas the DKT (OPT) and Specht (OPT) triangular elements appear to converge to a slightly lower value (4.26 vs. 4.31). Calculation times for the two finest mesh sizes are given in Table 22. We observe that the calculation times are approximately 50% higher for triangular elements, which is due to the larger number of quadrature points (a quadrilateral has 4 quadrature points, whereas when divided into two triangles it has $2\!\times \!3=6$ quadrature points).

**Table 18:** Sequential limit analysis of a simply supported plate under Case 1 boundary conditions evaluated at *λ=120* (triangle elements).

#elements	#dofs	SFELA step	Specht (OPT)	DKT (OPT)	DKMT (OPT)
$N_{e}$	$6N_{e}^2\!-\!4N_{e}\!+\!1$	[mm]	$u_Z(0,0)$ [mm]	$u_Z(0,0)$ [mm]	$u_Z(0,0)$ [mm]
$7\!\!\times \!\!7\!\!\times \!\!2$	267	0.0325	1.79132	1.75783	1.81404
$14\!\!\times \!\!14\!\!\times \!\!2$	1121	0.001625	1.77685	1.76522	1.77741
$28\!\!\times \!\!28\!\!\times \!\!2$	4593	0.0008125	1.76928	1.76811	1.77052
$56\!\!\times \!\!56\!\!\times \!\!2$	18593	0.00040625	1.76955	1.76926	1.76993

Reference solution: $u_{Z,\text {ref}}(0,0)=1.76986\,\text {mm}$ (Average of DKMQ24 and DKMQ24+ results, calculated on a $56\!\!\times \!\!56\!\!\times \!\!2$ mesh using a step size of 0.00040625 mm).

**Table 19:** Sequential limit analysis of a simply supported plate under Case 1 boundary conditions evaluated at *λ=120* (quad elements).

#elements	#dofs	SFELA step	DKQ (OPQ)	DKMQ (OPQ)	DKMQ24	DKMQ24+
$N_{e}$	$6N_{e}^2\!-\!4N_{e}\!+\!1$	[mm]	$u_Z(0,0)$ [mm]	$u_Z(0,0)$ [mm]	$u_Z(0,0)$ [mm]	$u_Z(0,0)$ [mm]
$7\!\!\times \!\!7$	267	0.0325	1.78322	1.78949	1.78487	1.77660
$14\!\!\times \!\!14$	1121	0.001625	1.76952	1.77304	1.76953	1.76951
$28\!\!\times \!\!28$	4593	0.0008125	1.76833	1.76934	1.76921	1.76874
$56\!\!\times \!\!56$	18593	0.00040625	1.76874	1.76962	1.77021	1.76952

Reference solution: $u_{Z,\text {ref}}(0,0)=1.76986\,\text {mm}$ (Average of DKMQ24 and DKMQ24+ results, calculated on a $56\!\!\times \!\!56$ mesh using a step size of 0.00040625 mm).

**Table 20:** Sequential limit analysis of a simply supported plate under Case 2 boundary conditions evaluated at *λ=120* (triangle elements).

#elements	#dofs	SFELA step	Specht (OPT)	DKT (OPT)	DKMT (OPT)
$N_{e}$	$6N_{e}^2\!-\!4N_{e}\!+\!1$	[mm]	$u_Z(0,0)$ [mm]	$u_Z(0,0)$ [mm]	$u_Z(0,0)$ [mm]
$7\!\!\times \!\!7\!\!\times \!\!2$	267	0.0325	4.25743	4.22025	4.36682
$14\!\!\times \!\!14\!\!\times \!\!2$	1121	0.001625	4.25846	4.24797	4.31205
$28\!\!\times \!\!28\!\!\times \!\!2$	4593	0.0008125	4.24258	4.25230	4.30819
$56\!\!\times \!\!56\!\!\times \!\!2$	18593	0.00040625	4.25749	4.25310	4.30790

Reference solution: $u_{Z,\text {ref}}(0,0)=4.31788\,\text {mm}$ (Average of DKMQ24 and DKMQ24+ results, calculated on a $56\!\!\times \!\!56\!\!\times \!\!2$ mesh using a step size of 0.00040625 mm).

**Table 21:** Sequential limit analysis of a simply supported plate under Case 2 boundary conditions evaluated at *λ=120* (quad elements). *-divergence.

#elements	#dofs	SFELA step	DKQ (OPQ)	DKMQ (OPQ)	DKMQ24	DKMQ24+
$N_{e}$	$6N_{e}^2\!-\!4N_{e}\!+\!1$	[mm]	$u_Z(0,0)$ [mm]	$u_Z(0,0)$ [mm]	$u_Z(0,0)$ [mm]	$u_Z(0,0)$ [mm]
$7\!\!\times \!\!7$	267	0.0325	3.60616*	4.09656	4.24768	4.28032
$14\!\!\times \!\!14$	1121	0.001625	3.60382*	4.27980	4.30094	4.30128
$28\!\!\times \!\!28$	4593	0.0008125	3.65222*	4.30051	4.30900	4.30590
$56\!\!\times \!\!56$	18593	0.00040625	3.66531*	4.31203	4.32386	4.31189

Reference solution: $u_{Z,\text {ref}}(0,0)=4.31788\,\text {mm}$ (Average of DKMQ24 and DKMQ24+ results, calculated on a $56\!\!\times \!\!56$ mesh using a step size of 0.00040625 mm).

**Table 22:** Calculation times for the two finest mesh sizes. This particular example was computed on a computer with an i7-14700HX CPU.

Element notation	Case 1 ($28\!\!\times \!\!28$)	Case 2 ($28\!\!\times \!\!28$)	Case 1 ($56\!\!\times \!\!56$)	Case 2 ($56\!\!\times \!\!56$)
	*t*[s]	*t*[s]	*t*[s]	*t*[s]
Specht (OPT)	1560	5040	15344	57180
DKT (OPT)	1406	4980	12429	59700
DKMT (OPT)	1022	4920	15784	59400
DKQ (OPQ)	826	3180	9905	36900
DKMQ (OPQ)	758	3180	10206	37740
DKMQ24	827	3600	10292	38520
DKMQ24+	751	3480	7789	38820

### Estimate of convergence orders

The convergence order *p* for all tested elements and test problems was estimated using the three finest mesh levels, with $p \approx -\frac{\log\frac{\varepsilon_k}{\varepsilon_{k-1}}}{\log\frac{N_{k}}{N_{k-1}}}-\frac{\log\frac{\varepsilon_{k-1}}{\varepsilon_{k-2}}}{\log\frac{N_{k-1}}{N_{k-2}}}$, where $N_k,\,N_{k-1},\,N_{k-2}$ denote the numbers of degrees of freedom, and $\varepsilon_k=\left|\frac{\lambda_k-\lambda_{\mathrm{ref}}}{\lambda_{\mathrm{ref}}}\right|,\,\varepsilon_{k-1}=\left|\frac{\lambda_{k-1}-\lambda_{\mathrm{ref}}}{\lambda_{\mathrm{ref}}}\right|,\,\varepsilon_{k-2}=\left|\frac{\lambda_{k-2}-\lambda_{\mathrm{ref}}}{\lambda_{\mathrm{ref}}}\right|$ denote the relative errors. Estimated orders of convergence are summarized in Table 23. In the case of the Ciria element and example 2, we observe higher convergence order due to adaptive refinement. Apart from this case, the highest convergence order was observed in example 1 for elements DKMQ (OPQ), DKMQ24, and DKMQ24+; in in-plane examples 2 and 5 for the OPQ element; and in examples 2 and 3 for the DKT (OPT) element.

**Table 23:** Estimate of convergence orders of the tested shell elements.

Element notation	Test problem				
	1	2	3	4	5
Bleyer	0.534	–	–	–	–
Ciria	–	1.485	–	–	–
Ciria (adaptive)	–	**2.695**	–	–	–
OPT	–	2.105	–	–	1.052
OPQ	–	**2.347**	–	–	**1.536**
Hodge/Belytschko	0.886	–	–	–	–
Makrodimopoulos (order 2)	0.962	–	–	–	–
Makrodimopoulos (order 3)	0.880	–	–	–	–
Makrodimopoulos (order 4)	0.836	–	–	–	–
T6b	1.000	–	–	–	–
Specht (OPT)	0.813	–	1.732	0.483	–
DKT (OPT)	0.670	–	**2.125**	**0.706**	–
DKQ (OPQ)	0.788	–	1.726	0.257	–
DKMT (OPT)	0.767	–	2.090	0.463	–
DKMQ (OPQ)	**1.056**	–	1.397	0.585	–
DKMQ24	**1.056**	1.525	1.319	0.672	1.382
DKMQ24+	**1.056**	1.613	1.857	0.484	0.625

Bold indicates the highest values.

## Conclusions

The DK and DKM classes of continuous shell elements underwent testing in a variety of FELA and SFELA benchmark problems (All elements were also tested on a variety of linear-elastic benchmarks). These benchmark problems incorporated different geometric models (including plates, membranes in the plane stress or plane strain approximation, and general shells) and different isotropic and orthotropic material models. The performance of these elements was compared with other elements from literature that are used in the context of FELA and SFELA. The main results are:

- The DK and DKM classes of continuous shell elements perform well in the context of FELA. These elements do not provide a rigid upper bound; however, they offer significantly lower discretization errors (see Figs. 5, 6, and 11 ) and higher convergence rates (see Table 23) compared to the triangular elements designed for the strict upper-bound^18,24,40^. Among all the tested shell elements, the quadrangles DKMQ24 and DKMQ24+ proved to be numerically most efficient for general shell problems, demonstrating neither shear nor membrane locking in the presented benchmarks.
- The lower numerical error of quadrangle elements over triangle elements is observed (see Figs. 5, 6, 11, 16, 22, 23, 29, 36).
- In the SFELA benchmark all the DK and DKM shell elements except for the DKQ (OPQ) quadrangle element coincide with the reference results given by the TRIC triangle. To replicate the reference results calculated with the TRIC triangle, the DKMQ (OPQ) quadrangle requires a finer mesh compared to other tested elements.
- However, for the DKM element class and the clamped plate problem, we encountered a loss of convergence on finer meshes if the ratio of the shell thickness to the shell characteristic length exceeds a certain limit. In this case, a numerical penalization was proposed to recover convergence.
- In all our test problems, convergence is monotonic: from below in one problem (the spherical cap) and from above in all other problems.

We can conclude that continuous shell elements of the DK and DKM classes, in particular the elements DKMQ24 and DKMQ24+, perform well in FELA and SFELA. They exhibit lower discretization error compared to triangular elements, which guarantee the strict upper bound. Moreover, the DKMQ24 and DKMQ24+ elements are computationally efficient with respect to the optimization time required, making them well-suited for practical implementations of FELA and SFELA. These advantages support their applicability to real-scale engineering problems, including large thin-walled structures, where both accuracy and computational efficiency are critical. Furthermore, the framework can be extended to materials exhibiting nonlinear hardening behavior, provided that the slope of the hardening curve tends to zero at infinity. In such cases, the hardening can be incorporated straightforwardly into the SFELA analysis. Future research could explore effect of adaptive meshing, which would further improve numerical efficiency of these shell elements.

## Data Availability

All data generated or analysed during this study are included in this published article.

## Appendix 1: Derivation of the dissipation power density for the considered stress-based failure criteria

### Plane-stress Tresca failure criterion

The Tresca in-plane plane stress failure criterion is given by:

52 $$\begin{aligned} \max (|\sigma _2-\sigma _1|,|\sigma _1|,|\sigma _2|)\le \sigma _0, \end{aligned}$$

where $\sigma _1, \sigma _2$ are eigenstresses. The dissipation power density is defined by:

53 $$\begin{aligned} d_{\text {p}}^{z}(\dot{\boldsymbol{\epsilon }})&=\max _{\forall \boldsymbol{\sigma }:~\!\max (|\sigma _2-\sigma _1|,|\sigma _1|,|\sigma _2|)\le \sigma _0}~\sigma _1\dot{\epsilon }_1+\sigma _2\dot{\epsilon }_2. \end{aligned}$$

The expression $\sigma _1\dot{\epsilon }_1+\sigma _2\dot{\epsilon }_2$ is linear (for given strain rates $\dot{\epsilon }_1,\dot{\epsilon }_2$) and therefore the maximum can be found only at boundary points: $[0,\pm \sigma _0],~[\pm \sigma _0, 0],~\pm [\sigma _0, \sigma _0]$. This immediately yields:

54 $$\begin{aligned} d_{\text {p}}^{z}(\dot{\boldsymbol{\epsilon }})&=\sigma _0\max \left( |\dot{\epsilon }_1|, |\dot{\epsilon }_2|, |\dot{\epsilon }_1+\dot{\epsilon }_2|\right) . \end{aligned}$$

By using standard results for eigenvectors in 2D, the final expression of the dissipation power density is obtained:

55 $$\begin{aligned} d_{\text {p}}^z(\dot{\boldsymbol{\epsilon }})&=\sigma _0\max \left[ \left| \frac{\dot{\epsilon }_x+\dot{\epsilon }_y-\sqrt{A}}{2}\right| , \left| \frac{\dot{\epsilon }_x+\dot{\epsilon }_y+\sqrt{A}}{2}\right| ,|\dot{\epsilon }_x+\dot{\epsilon }_y|\right] , \end{aligned}$$

56 $$\begin{aligned} A&=(\dot{\epsilon }_x - \dot{\epsilon }_y)^2+\dot{\curlyvee }_{xy}^2. \end{aligned}$$

The expression (55) can be easily implemented in the SOCP framework, where *A* is calculated as a cone and each absolute value is prescribed by two linear inequalities (without an explicit use of absolute values).

### Plane-strain Tsai-Wu failure criterion

The plane-strain Tsai-Wu failure criterion is given by the yield function:

57 $$\begin{aligned}&f=\sigma _x\left[ \frac{1}{f_{\text {t}x}}-\frac{1}{f_{\text {c}x}}\right] + (\sigma _y+\sigma _z)\left[ \frac{1}{f_{\text {t}y}}-\frac{1}{f_{\text {c}y}}\right] + \frac{\sigma _x^2}{f_{\text {t}x}f_{\text {c}x}}+\frac{\sigma _y^2+\sigma _z^2}{f_{\text {t}y}f_{\text {c}y}} +\frac{\tau _{xy}^2}{f_{\text {v}xy}^2} \le 1. \end{aligned}$$

The dissipation power density is defined by:

58 $$\begin{aligned} d_{\text {p}}^z(\dot{\boldsymbol{\epsilon }})=&\max _{f(\boldsymbol{\sigma })\le 1}~\sigma _x\dot{\epsilon }_x+\sigma _y\dot{\epsilon }_y+\tau _{xy}\dot{\curlyvee }_{xy}. \end{aligned}$$

The Lagrangian and its derivatives yield:

59 $$\begin{aligned} {\mathcal {L}}&=\sigma _x\dot{\epsilon }_x+\sigma _y\dot{\epsilon }_y+\tau _{xy}\dot{\curlyvee }_{xy}+\lambda f, \end{aligned}$$

60 $$\begin{aligned} \frac{\partial {\mathcal {L}}}{\partial \tau _{xz}}&=\frac{2\tau _{xz}}{f_{\text {v}xy}^2}\lambda =0~\implies ~\tau _{xz}=0,~~~~ \frac{\partial {\mathcal {L}}}{\partial \tau _{yz}}=\frac{2\tau _{yz}}{f_{\text {v}xy}^2}\lambda =0~\implies ~\tau _{yz}=0,\end{aligned}$$

61 $$\begin{aligned} \frac{\partial {\mathcal {L}}}{\partial \tau _{xy}}&=\dot{\curlyvee }_{xy}+\frac{2\tau _{xy}}{f_{\text {v}xy}^2}\lambda =0~\implies ~\tau _{xy}=-\frac{\dot{\curlyvee }_{xy}f_{\text {v}xy}^2}{2\lambda },\end{aligned}$$

62 $$\begin{aligned} \frac{\partial {\mathcal {L}}}{\partial \sigma _x}&=\dot{\epsilon }_x+\lambda \left( \frac{1}{f_{\text {t}x}}-\frac{1}{f_{\text {c}x}}+\frac{2\sigma _x}{f_{\text {t}x}f_{\text {c}x}}\right) =0~\implies ~\sigma _x=\frac{f_{\text {t}x}-f_{\text {c}x}}{2}-\frac{f_{\text {t}x}f_{\text {c}x}}{2\lambda }\dot{\epsilon }_x,\end{aligned}$$

63 $$\begin{aligned} \frac{\partial {\mathcal {L}}}{\partial \sigma _y}&=\dot{\epsilon }_y+\lambda \left( \frac{1}{f_{\text {t}y}}-\frac{1}{f_{\text {c}y}}+\frac{2\sigma _y}{f_{\text {t}y}f_{\text {c}y}}\right) =0~\implies ~\sigma _y=\frac{f_{\text {t}y}-f_{\text {c}y}}{2}-\frac{f_{\text {t}y}f_{\ cy}}{2\lambda }\dot{\epsilon }_y,\end{aligned}$$

64 $$\begin{aligned} \frac{\partial {\mathcal {L}}}{\partial \sigma _z}&=\lambda \left( \frac{1}{f_{\text {t}y}}-\frac{1}{f_{\text {c}y}}+\frac{2\sigma _z}{f_{\text {t}y}f_{\text {c}y}}\right) =0~\implies ~\sigma _z=\frac{f_{\ ty}-f_{\text {c}y}}{2}. \end{aligned}$$

By inserting all stresses into a function *f* (Eq.(57)), we get the expression for *λ*:

65 $$\begin{aligned} 2\lambda =\pm \sqrt{ \frac{f_{\text {t}x}f_{\text {c}x}(\dot{\epsilon }_x)^2+ f_{\text {t}y}f_{\text {c}y}(\dot{\epsilon }_y)^2+ (f_{\text {v}xy}\dot{\curlyvee }_{xy})^2}{\chi }},~\chi =1+\frac{1}{4}\frac{(f_{\text {t}x}-f_{\text {c}x})^2}{f_{\text {t}x}f_{\text {c}x}}+\frac{1}{2}\frac{(f_{\text {t}y}-f_{\text {c}y})^2}{f_{\text {t}y}f_{\text {c}y}}, \end{aligned}$$

where the minus sign is chosen according to the positiveness of $d_{\text {p}}^z(\dot{\boldsymbol{\epsilon }})$. The final expression of the dissipation power density has the form:

66 $$\begin{aligned} d_{\text {p}}^z(\dot{\boldsymbol{\epsilon }})=&\frac{f_{\text {t}x}-f_{\text {c}x}}{2}\dot{\epsilon }_x+\frac{f_{\text {t}y}-f_{\text {c}y}}{2}\dot{\epsilon }_y + \sqrt{(\boldsymbol{R}^{-\text {T}}\dot{\boldsymbol{\epsilon }})^{\text {T}}\boldsymbol{R}^{-\text {T}}\dot{\boldsymbol{\epsilon }}}, \end{aligned}$$

where

67 $$\begin{aligned} \boldsymbol{R}^{-\text {T}}=&\left[ \begin{array}{ccc} \sqrt{\chi f_{\text {t}x}f_{\text {c}x}} & 0 & 0\\ 0 & \sqrt{\chi f_{\text {t}y}f_{\text {c}y}} & 0\\ 0 & 0 & \sqrt{\chi }f_{\text {v}xy}\\ \end{array}\right] ,~\chi =1+\frac{1}{4}\frac{(f_{\text {t}x}-f_{\text {c}x})^2}{f_{\text {t}x}f_{\text {c}x}}+\frac{1}{2}\frac{(f_{\text {t}y}-f_{\text {c}y})^2}{f_{\text {t}y}f_{\text {c}y}}. \end{aligned}$$

## Appendix 2: Practical numerical experiences with optimization

As the optimizer we used MOSEK version 10.1.27^5^. As an input to MOSEK a script file in the *opf* file format was used. The following suggestions help to achieve calculation efficiency and accuracy:

1. We eliminate zero dofs of $\dot{\boldsymbol{\mathfrak {q}}}$ from the primal formulation (P), rather than just setting them to zero.
2. We eliminate as many variables as possible from the formulation if their values are not explicitly needed. For example, eliminating $\boldsymbol{m}_{eq}$ in Eqs. (29–32) leads to Eqs.(33–35).
3. We use parallelization for creating the *opf* script file.
4. We use parallelization in MOSEK with the setting [p MSK_IPAR_NUM_THREADS] 16 [/p]. Additionally, we use the setting [p MSK_IPAR_INTPNT_SOLVE_FORM] MSK_SOLVE_PRIMAL [/p].
5. It is important to remember that round-off errors can disrupt convergence if the primal variables differ by many orders of magnitude, which can easily occur in practice. MOSEK automatically handles the scaling between primal and dual variables, but the scaling of the primal variables is left to the user. Therefore, we scale the problem variables by reducing plastic strengths by *n* orders of magnitude to keep the underlying optimization problem well-conditioned. Consequently, the resulting plastic multiplier *λ* must be increased by *n* orders of magnitude. In our case, we use *n=9* in test problem 5 (block with asymmetric holes) and *n=6* in all other test problems.
6. It is beneficial to use the dual formulation (D) instead of the primal one.

## Abbreviations

c.s.: Coordinate system
dofs: Degrees of freedom
FELA: Finite-element-based limit analysis
SFELA: Sequential finite-element-based limit analysis
SOCP: Second order cone programming

## Latin symbols

$C_{\text {d}}$: Penalty constant for drilling rotations stabilization
$d_{\text {p}}^{z}$: Dissipation power density
$d_{\text {p}}$: Dissipation power area-density
$$D_{\text {p}}$$: Dissipation power
$$E,E_x, E_y$$: Young’s moduli
*f*: Failure criterion
$\sigma _0$: Yield strength in the isotropic case
$f_{\text {t}x},~f_{\text {t}y}$: Yield strengths in tension
$f_{\text {c}x},~f_{\text {c}y}$: Yield strengths in compression
$f_{\text {v}xy}$: Yield strength in shear in *xy* plane
$\boldsymbol{\mathfrak {f}}$: Discretized loading vector
*g*: Gravitational acceleration
$$G_{xy}$$: Shear modulus
*h*: Shell thickness
$m_{\text {p}}=\sigma _0\frac{h^2}{4}$: Maximum plastic moment
*n*: Number of element nodes
$n_{\text {p}}=\sigma _0h$: Maximum plastic in-plane force
$N_{\text {opt}}$: Total number of optimization unknowns
$\#\text {dofs}$: Total number of kinematic (nodal) dofs
$N_{d}$: Total number of nodes
$N_{e}$: Total number of elements
$N_{\text {edge}}$: Total number of element edges
$N_q$: Number of quadrature points in an element
$N_r$: Number of quadrature points in *z*-direction
*p*: Pressure
$\dot{\boldsymbol{q}} = {\left[ \dot{\boldsymbol{\varphi }},~ \dot{\boldsymbol{u}} \right] }^{\text {T}}$: Vector of velocities at a given point
$\dot{\boldsymbol{\mathfrak {q}}}$: Vector of all unknown velocities
$\dot{u}_x,\dot{u}_y,\dot{u}_z$: Nodal velocities in the local c.s.
$\dot{u}_X,\dot{u}_Y,\dot{u}_Z$: Nodal velocities in the global c.s.
$w_i$: *i*-th Quadrature weight
*xyz*: Coordinates of the local c.s.
*XYZ*: Coordinates of the global c.s.

## Greek symbols

*Γ*: *Ω* Boundary
$\dot{\epsilon }_x, \dot{\epsilon }_y, \dot{\curlyvee }_{xy}$: In-plane plastic strain rates
$\dot{\epsilon _1},\dot{\epsilon _2}$: In-plane eigenstrain rates
$\overline{\varepsilon }^{\text {p}}$: Accumulated plastic strain
$\dot{\varepsilon }_x, \dot{\varepsilon }_y, \dot{\gamma }_{xy}$: Membrane plastic strain rates
$\dot{\boldsymbol{{\mathcal {E}}}}={[\dot{\boldsymbol{\kappa }},\dot{\boldsymbol{\varepsilon }}]}^{\text {T}}$: Vector of plastic strain rates
$\dot{\kappa }_x, \dot{\kappa }_y, \dot{\kappa }_{xy}$: Bending plastic strain rates
*λ*: Plastic load multiplier
$$\nu , \nu _{xy}$$: Poisson’s ratii
$\boldsymbol{\sigma }={[\sigma _x,\sigma _y,\tau _{xy}]}^{\text {T}}$: In-plane stress
$\sigma _1,\sigma _2$: In-plane eigenstresses
$\dot{\varphi }_x,\dot{\varphi }_y,\dot{\varphi }_z$: Nodal rotational velocities in the local c.s.
$\dot{\varphi }_X,\dot{\varphi }_Y,\dot{\varphi }_Z$: Nodal rotational velocities in the global c.s.
*Ω*: Shell mid-surface
